# Efficient and rapid conversion of human astrocytes and ALS mouse model spinal cord astrocytes into motor neuron-like cells by defined small molecules

**DOI:** 10.1186/s40779-020-00271-7

**Published:** 2020-09-06

**Authors:** An-Dong Zhao, Hua Qin, Meng-Li Sun, Kui Ma, Xiao-Bing Fu

**Affiliations:** 1 Tianjin Medical University, Tianjin, 300070 China; 2 Research Center for Tissue Repair and Regeneration affiliated to the Medical Innovation Research Division and 4th Medical Center, PLA General Hospital and PLA Medical College, 28 Fu Xing Road, Haidian District, Beijing, 100853 P. R. China; 3 PLA Key Laboratory of Tissue Repair and Regenerative Medicine and Beijing Key Research Laboratory of Skin Injury, Repair and Regeneration, Beijing, 100048 China; 4 Research Unit of Trauma Care, Tissue Repair and Regeneration, Chinese Academy of Medical Sciences, Beijing, 100048 China

**Keywords:** Human astrocyte, Motor neuron, Reprogramming, Transdifferentiation, Conversion, Regeneration, Amyotrophic lateral sclerosis, Neurodegenerative

## Abstract

**Background:**

Motor neuron degeneration or loss in the spinal cord is the characteristic phenotype of motor neuron diseases or spinal cord injuries. Being proliferative and located near neurons, astrocytes are considered ideal cell sources for regenerating neurons.

**Methods:**

We selected and tested different combinations of the small molecules for inducing the conversion of human and mouse astrocytes into neurons. Microscopic imaging and immunocytochemistry analyses were used to characterize the morphology and phenotype of the induced neurons while RT-qPCR was utilized to analyze changes in gene expression. In addition, whole-cell patch-clamp recordings were measured to examine the electrophysiological properties of induced neurons.

**Results:**

The results showed that human astrocytes could be rapidly and efficiently converted into motor neuron-like cells by treatment with defined small molecules, with a yield of over 85% motor neuron-like cells attained. The induced motor neuron-like cells expressed the pan-neuronal markers TUJ1, MAP2, NeuN, and Synapsin 1 and motor neuron markers HB9, ISL1, CHAT, and VAChT. During the conversion process, the cells did not pass through a proliferative neural progenitor cell intermediate. The induced motor neurons were functional, showing the electrophysiological properties of neurons. The same chemical cocktail could induce spinal cord astrocytes from an amyotrophic lateral sclerosis mouse model carrying a SOD1 mutation to become motor neuron-like cells that exhibited a decrease in cell survival and an increase in oxidative stress compared to that observed in wild-type MNs derived from healthy mice. Moreover, the chemical induction reduced oxidative stress in the mutant astrocytes.

**Conclusion:**

The results of the present study demonstrated the feasibility of chemically converting human and mouse astrocytes into motor neuron-like cells that are useful for neurodegenerative disease modeling and regenerative medicine.

## Background

Motor neurons (MNs) are a specialized type of neurons residing in spinal cords that innervate skeletal muscles and control their movement. MN degeneration or loss in diseases such as spinal muscular atrophy, amyotrophic lateral sclerosis (ALS), and spinal cord injury (SCI) can result in paralysis or death [1]. Currently, there are no effective treatments for these motor neuron diseases [1]. Thus, there is a growing interest in generating MNs for cell replacement therapy and in vitro disease modeling to elucidate the mechanisms underlying MN degeneration. Considerable progress has been made in generating MNs from pluripotent stem cells, including embryonic stem cells (ESCs) and induced pluripotent stem cells (iPSCs) for which variety of differentiation protocols have been developed: a series of induction steps, including embryoid body (EB) induction, neural rosette formation, neural patterning, and neuronal maturation [2–5]. Though previous studies have improved the differentiation protocols using small molecules [6–10], these methods are tedious, time-consuming (1 to 2 months), inefficient (30–70%), or require genetic manipulations.

Direct lineage conversion can reprogram somatic cells into functional neurons without passing through a pluripotent intermediate. For instance, the direct conversion of somatic cells (e.g., fibroblasts) into MNs has been achieved by viral-based expression of transcription factors in several laboratories [11–14]. However, the potential risks of tumorigenesis and the difficulty in delivering exogenous genes in vivo limit their clinical applications.

In our previous studies, we developed a small molecule-based method to convert human fibroblasts into MN-like cells [15]. The use of small, cell-permeable molecules has emerged as a novel strategy to induce direct lineage conversion, generating cardiomyocytes, neural progenitor cells, and neurons from fibroblasts [16–19]. Compared with transcription factor-based lineage conversion, the small molecule-based conversion approach may be potentially translated into therapeutic applications.

Astrocytes, the most abundant cell types in spinal cords, play important roles in maintaining homeostasis and modulating neural circuit activity. In response to injuries, astrocytes proliferate, form scars at lesion sites, and subsequently impair endogenous neuron regeneration. Therefore, reprogramming astrocytes into neurons may be a promising strategy to promote neuron regeneration. Previous studies have reported that mouse and human astrocytes could be reprogrammed into neurons by forced expression of transcription factors or small molecules [20–23]. However, the induced neurons are not MNs. Whether astrocytes can be induced to cells functioning as MNs using small molecules awaits further investigation.

In the present study, we identified a defined cocktail of small molecules that can directly convert human astrocytes into MN-like cells, without passing through a neural progenitor cell stage. In addition, the same cocktails could reprogram spinal cord astrocytes derived from ALS mouse models into MN-like cells that displayed a decrease of cell survival capability and an increase of oxidative stress level compared to that observed in wild-type MNs derived from healthy mice. Thus, the results of our study showed the feasibility of chemically converting human and mouse astrocytes into MN-like cells that are useful for neurodegenerative disease modeling and regenerative medicine.

## Methods

### Human astrocyte culture

Human astrocytes (HA1800) were purchased from ScienCell (CA, USA) and were cultured and maintained in a growth medium comprising GibcoDulbecco’s Modified Eagle Medium: F-12 (DMEM/F12, GIBCO) supplemented with 10% fetal bovine serum (FBS, GIBCO) and 1% penicillin/streptomycin (GIBCO). Cells cultured to greater than 90% confluence were dissociated using TrypLE (GIBCO), centrifuged at 1000 rpm for 5 min, resuspended, and replated in fresh growth medium. Cells were maintained at 37 °C under a humidified atmosphere of air and 5% CO_2_.

### Reprogramming human astrocytes into neurons

Human astrocytes were plated on 15-mm coverslips coated with Matrigel (BD Biosciences) at 50,000 cells per coverslip in 24-well plates (Corning). Cells were cultured in human astrocyte growth medium until reaching 90% confluence. Then, the culture medium was replaced with induction medium comprising neurobasal medium (GIBCO) supplemented with 0.5% N2 (GIBCO), 1% B27 (GIBCO), and 1% penicillin/streptomycin as well as small molecules in the test group or 1% DMSO in the control group. The following small molecules were used at the indicated concentrations: 5 μM kenpaullone (MedChem Express #HY-12302), 10 μM forskolin (Selleck #S2449), 10 μM Y-27632 (Selleck #S1049), 2 μM purmorphamine (MedChem Express #HY-15108), and 2 μM retinoic acid (Sigma #R2625). The induction medium plus the small molecules were refreshed every 2 days. On day 7, the culture medium was replaced with neuron maturation medium comprising neurobasal medium supplemented with 0.5% N2, 1% B27, 20 ng/ml of brain-derived neurotrophic factor (BDNF, PeproTech), 20 ng/ml of glial cell-derived neurotrophic factor (GDNF, PeproTech), 10 ng/ml of NT3 (PeproTech) and 10 μM forskolin. Half of the maturation medium was replaced with fresh medium every other day. Other small molecules used in the study included valproic acid (VPA, 0.5 mM, MedChem Express #HY-10585), Respsox (5 μM, MedChem Express #HY-13012), SB43152 (5 μM, Selleck #S1067), N-[N-(3,5-Difluorophenacetyl)-L-alanyl]-S-phenylglycine t-butyl ester (DAPT, 5 μM, MedChem Express #HY-13027), dorsomorphin (1 μM, MedChem Express #HY-13418A), LDN193189 (0.1 μM, Selleck #DM3189), and CHIR99021 (5 μM, MedChem Express, #HY-10182).

### BrdU incorporation assay

Human astrocytes were incubated with 5-bromo-2-deoxyuridine (BrdU, Millipore) at a final concentration of 10 μM for 4 h and then treated with small molecules. In another group, cells were pulse-labeled with 10 μM BrdU for 2 h before being evaluated through immunocytochemical analysis at 24 and 48 h after treatment with small molecules.

### Isolation of spinal cord astrocytes and MNs from ALS mice

All experimental procedures and protocols were performed in accordance with the recommendations of the National Institutes of Health Guidelines for the Care and Use of Laboratory Animals and approved by the Animal Experimentation Ethics Committee of the Fourth Medical Center of PLA General Hospital. Female B6SJL-Tg(SOD1*G93A)/Nju mice aged 8 weeks old were purchased from the Model Animal Research Center of Nanjing University, and female C57BL/6 J mice aged 8 weeks old were purchased from SPF Biotechnology Co. The isolation of spinal cord astrocytes and MNs from adult mice was performed as described previously [24], with some modifications. Briefly, the vertebral columns of mice were separated using straight scissors, followed by extruding the adult spinal cords with a syringe containing cold medium into cold growth medium (DMEM/F12 supplemented with 20% FBS and 1% penicillin/streptomycin). The collected spinal cords were cut into small pieces, digested by 2 mg/ml papain (dissolved in PBS) for 30 min and then mechanically dissociated to produce single cells. For astrocyte culture, cells were centrifuged at 500×g for 10 min at 4 °C. For MN culture, cells were centrifuged at 280×g for 10 min at 4 °C. The astrocyte pellets were resuspended in growth medium and plated on poly-D-lysine-coated T25 flasks and incubated at 37 °C under an atmosphere of 5% CO_2_ and 95% air. When cells grew to confluence (10–12 d), the culture flasks were shaken on a rotary shaker at 280 rpm for 20 h at 37 °C to remove the loosely attached microglia. Then, the attached astrocytes were detached by trypsin and subjected to chemical induction.

The MN pellets were resuspended in cold neurobasal medium, laid over a NycoPrep™ 1.077 density solution (PROGEN) and then centrifuged at 900×g for 20 min at 4 °C. MNs were collected at the interface of the NycoPrep density solution and then transferred into a new 50 ml collection tube which was filled with cold neurobasal medium and centrifuged at 425×g for 10 min. Then, the cell pellets were resuspended with the neurobasal medium supplemented with 1% N2, 0.5% B27, 2 mM GlutaMAX™, 1% penicillin/streptomycin, 20 ng/ml BDNF, 20 ng/ml GDNF, and 10 ng/ml NT3 and then cultured on the Matrigel-coated plates.

### Chemical induction of mouse astrocytes

ALS astrocytes were seeded on Matrigel-coated coverslips. ALS astrocytes were induced using small molecules (5 μM kenpaullone, 10 μM forskolin, 10 μM Y-27632, 2 μM purmorphamine, 2 μM retinoic acid) in the neurobasal medium and then maintained in the neuron maturation medium. After chemical induction, the induced neurons were characterized in immunostaining assay and other experiments.

### Neurosphere formation assay

Neurosphere formation was analyzed for human astrocytes and hNPCs by seeding 1 × 10^4^ cells in the wells of ultralow-attachment six-well plate. The growth medium for neurosphere formation was neurobasal medium supplemented with 0.5% N2, 1% B27, 20 ng/ml EGF (Invitrogen), 20 ng/ml basic fibroblast growth factor (bFGF, PeproTech), and 1% penicillin/streptomycin. The growth medium was changed each day by spinning down the cells at 1000 rpm for 5 min and then resuspending them in fresh growth medium with images of cells captured 7 days later.

### Neuronal differentiation assay

To investigate whether NPCs were present among the cultured human astrocytes, we cultured human astrocytes in neuronal differentiation medium comprising neurobasal medium supplemented with 0.5% N2, 1% B27, 20 ng/ml BDNF, 20 ng/ml GDNF, 20 ng/ml IGF, 20 ng/ml NT3, 20 μM L-ascorbic acid, 2 μM cAMP and 1% penicillin/streptomycin. The medium was exchanged every 2 days and after 4 weeks, the cultured cells were evaluated in immunostaining assays.

### Immunocytochemistry

For immunocytochemical staining, cells plated on glass coverslips were fixed with 4% formaldehyde (PFA) for 10 min at room temperature (RT). Tissue samples were fixed with cold acetone for 10 min at 4 °C. Then, glass coverslips and tissue samples were washed 3 times with phosphate-buffered saline (PBS) and incubated in blocking buffer (5% goat serum, 1% bovine serum albumin, and 0.5% Triton X-100) for 30 min at RT. Subsequently, the samples were incubated with primary antibodies at 4 °C overnight, washed 3 times with PBS, and then incubated with the appropriate fluorescent probe-conjugated secondary antibodies for 1 h at RT. Cell nuclei were counterstained with DAPI. Images were captured under a fluorescence microscope (Olympus) or Leica Sp8 confocal microscope. The following primary antibodies were used: rabbit anti-GFAP (1:500, Abcam), mouse anti-GFAP (1:100, Santa Cruz), mouse anti-TUJ1 (1:500, Covance), rabbit anti-TUJ1(1:500, Sigma), mouse anti-O4 (1:200, Millipore), rabbit-anti MAP2 (1:500, Millipore), mouse anti-HB9 [1:50, 81.5C10, Developmental Studies Hybridoma Bank (DSHB)], rabbit anti-HB9 (1:200, Abcam), mouse anti-ISL1 (1:100, DSHB), rabbit anti-ISL1 (1:200, Abcam), goat anti-CHAT (1:200, Millipore), rabbit anti-VAChT (1:1000, SYSY), rabbit ani-GABA (1:500, Sigma), rabbit anti-vGlut1 (1:1000, Invitrogen), mouse anti-TH (1:200, Millipore), mouse anti-NeuN (1:200, Millipore), rabbit anti-SYN (1:500, Millipore), rabbit anti-SOX2 (1:200, Millipore), mouse anti-NESTIN (1:200, Millipore), rabbit anti-PAX6 (1:500, Biolegend), rabbit anti-Ki67 (1:500, ab15580, Abcam), and mouse anti-BrdU (1:50, 2750, Millipore). The Alexa 488 and Alexa 594-conjugated secondary antibodies were obtained from either Jackson Immunoresearch Laboratories or Invitrogen.

### Time-lapse imaging

Human astrocytes were labeled by transfection with the lentiviral vector pLenti-EGFP:T2A (OBiO Technology, Shanghai, China) expressing EGFP (green) and then treated with small molecules. During the chemical induction, cells were imaged by confocal microscopy in the time-lapse live cell imaging mode.

### Neuronal conversion efficiency

The neuronal conversion efficiency was calculated as previously described, with some minor modifications [19, 25]. Briefly, 10 randomly selected 20× visual fields were used to count cell numbers. The total number of TUJ1^+^, TUJ1^+^HB9^+^, TUJ1^+^ISL1^+^, and TUJ1^+^CHAT^+^ cells were determined and the neuronal conversion efficiency was calculated as the percentages of TUJ1^+^ cells among the total DAPI^+^ cells. The conversion efficiency and purity of induced MN-like cells were calculated as the percentage of TUJ1^+^HB9^+^, TUJ1^+^ISL1^+^, or TUJ1^+^CHAT^+^ cells relative to the total percentage of DAPI^+^ or total TUJ1^+^ cells. The data are presented as the means ± SEM from triplicate samples.

### Measurement of neurite length

To measure changes in the neurite length in human astrocytes induced by the combination of small molecules including Kenpaullone, Forskolin, Y-27632, Purmorphamine, and RA (KFYPR), CFYPR (CHIR99021+FYPR), and FYPR, we stained the induced cells with TUJ1 antibodies. The fluorescent images were captured under a confocal microscope with the same magnification 20× at the same time. As TUJ1 was expressed in the cytosol of neurons, the neurite length was calculated by TUJ1 immunostaining from the cell body to the end of the neurite by the ImageJ software with the NeuronJ plugin. The data were presented as the ratio of neurite lengths of KFYPR-induced cells or FYPR-induced cells to that of CFYPR-induced cells.

### Electrophysiology analysis

Whole-cell patch-clamp recordings of neurons were performed at room temperature using an inverted microscope and an EPC-10 amplifier (HEKA). Cells in different groups were maintained in a bath solution of 150 mM NaCl, 4 mM KCl, 2 mM MgCl_2_, 2 mM CaCl_2_, 10 mM HEPES, and 10 mM glucose (pH 7.4, 300 mOsm). Patch pipettes were pulled and polished to yield a resistance of 3–4 MΩ when filled with the intracellular solution [130 mM K-gluconate, 6 mM KCl, 3 mM NaCl, 0.2 mM EGTA, 10 mM HEPES, 4 mM ATP-Mg, 0.4 mM GTP-Na, and 14 mM phosphocreatine-di(Tris) (pH 7.2, 285 mOsm)]. During recordings, the pipette capacitance was neutralized, and access resistance was continuously monitored. In the current-clamp recording mode, action potentials were elicited by a depolarizing step current from − 60 pA to 120 pA at 20pA increments and 800 ms in duration. In the voltage-clamp mode, whole-cell currents were evoked with voltage steps ranging from − 60 to 30 mV at 10-mV increments. Then, the tetrodotoxin (TTX, 1 μM) was applied to the chamber, and the voltage steps were repeated to examine TTX-sensitive currents. Currents were filtered and digitized at 3 and 10 kHz, respectively, and the data were analyzed using pClamp 9.0 (Axon Instruments).

### Quantitative reverse Ftranscription polymerase chain reaction (qRT-PCR)

Total RNA was extracted from the indicated cell samples using TRIzol (Invitrogen, USA) following the manufacturer’s instructions and was reverse-transcribed by using the High Capacity cDNA Reverse Transcription Kit (Applied Biosystems). Quantitative reverse transcription PCR involved the use of SYBR Premix Ex Taq II (Takara) in a 7300 Real-time PCR system (Applied Biosystems). The relative expression levels were normalized to that of the internal control (GAPDH). All primer sequences are listed in Additional file 5.

### Flow cytometry

HA1800 astrocytes were dissociated into single cells using TrypLE and then washed with cold PBS containing 0.5% BSA and 2 mM EDTA. Subsequently, the astrocytes were incubated with FITC-conjugated mouse anti-human CD44 or FITC-conjugated mouse IgG2a, κ isotype control (BD Bioscience, USA) for 30 min at 4 °C in the dark before being washed 3 times with cold PBS buffer. FASCalibur (BD Biosciences) and Flowjo software were used to acquire and analyze data.

### Detection of reactive oxygen species (ROS) production

An image-IT™ LIVE Green Reactive Oxygen Species Detection Kit was used to detect the total cellular ROS according to the manufacturer’s instructions (Invitrogen). Briefly, the assay is based on the use of live cell-permeable 5-(and-6)-carboxy − 2′,7-dichlorodihydrofluorescein diacetate (carboxy-H2DCFDA), a compound that can emit green fluorescence when oxidized by ROS. The ROS production level was measured by counting the number of fluorescent cells in 10 randomly selected 20× view fields under a microscope.

### Lactate dehydrogenase (LDH) activity assay

LDH activity assays were performed as described previously [26]. Briefly, the supernatants from the cultures of ALS-As-iMNs and wild-type MNs were collected after a 4-week culture. Then, the supernatants were centrifuged, and the proteins in the supernatants were concentrated with protein concentrators (Thermo Scientific™). The LDH activity was measured in the concentrated supernatants with the LDH-cytotoxicity colorimetric assay kit II (BioVision). The genomic DNA (gDNA) in ALS-As-iMNs and wild-type MNs was extracted using the genomic DNA purification kit (Thermo Scientific™). The LDH activity values were normalized to the amount of gDNA of the ALS-As-iMNs and wild-type MNs.

### Statistical analysis

All quantified data were statistically analyzed and are presented as means ± SEM. Statistical significance of differences between groups was determined by Student’s *t*-test and a *P*-value < 0.05 was considered significant.

## Results

### Characterization of cultured human astrocytes

**Fig. 1:**
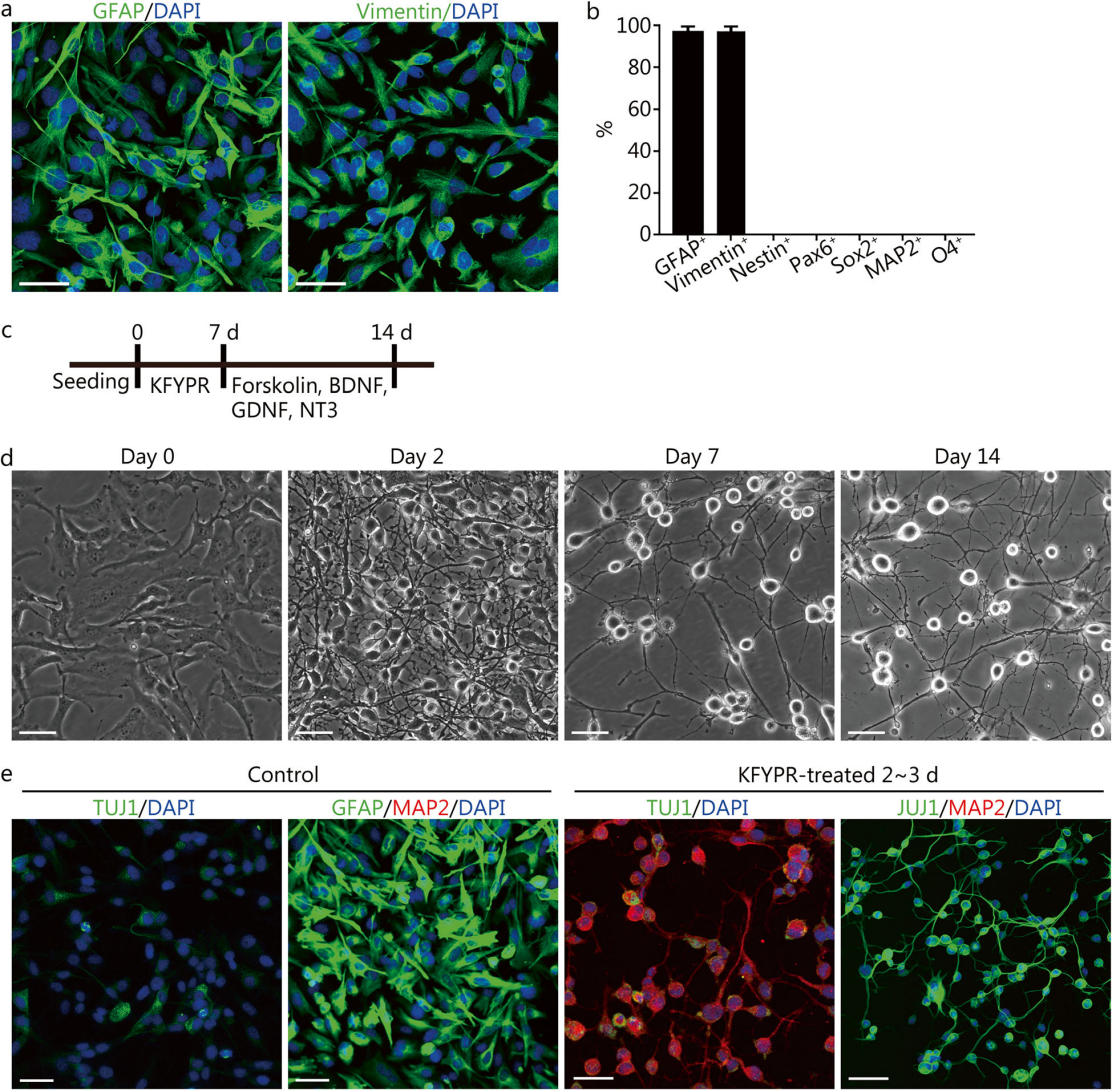
Characterization of human astrocytes and the phenotypic conversion after chemical induction. **a** Immunostaining of cultured human astrocytes for the astrocyte markers glial fibrillary acidic protein (GFAP) and Vimentin. **b** Quantitative analysis of cultured human astrocytes for marker expression of astrocytes, neural progenitor cells (NPCs), neuronal cells, or oligodendrocytes. The data are presented as the means ± SEM from three independent experiments. **c** A schematic diagram showing the small molecule-based induction protocol. K, kenpaullone; F, forskolin; Y, Y-27632; P, purmorphamine; R, retinoic acid. **d** Microscopic images of the cell morphological changes after chemical induction. **e** Expression of class III β-tubulin 1 (TUJ1), microtubule-associated protein 2 (MAP2), and GFAP in the control group and the KFYPR-induced group. All scale bars = 50 μm

In the present study, we used human astrocytes (HA1800, ScienCell) for the chemical reprogramming assays. To avoid the presence of neural progenitor cells (NPCs), we cultured and maintained human astrocytes in the culture medium containing 10% FBS, which stimulates the differentiation of NPCs. Indeed, the cultured human astrocytes did not express NPC markers (NESTIN, PAX6, and SOX2) and were unable to form neurospheres in the neuronal differentiation medium (Additional file 1a and d and Fig. 1b), whereas human NPCs (hNPCs) expressed NPC markers and formed neurospheres (Additional file 1b and d). Immunostaining analysis showed that cultured human astrocytes were immunopositive for the astrocyte markers glial fibrillary acidic protein (GFAP; 99%) and Vimentin (98%) (Fig. 1a and b). The flow cytometry results revealed that approximately 99% of the cultured cells expressed CD44 (Additional file 1c), which is expressed by human astrocytes but not hNPCs or neuronal cells. In addition, the cultured cells were negative for neuronal markers class III β-tubulin 1 (TUJ1), microtubule-associated protein 2 (MAP2) and oligodendrocyte marker O4 (Additional file 1b and b). These results demonstrate that our cultured cells are without detectable contamination by NPCs or other glial cells.

### Direct conversion of human astrocytes into MN-like cells by defined small molecules

**Fig. 2:**
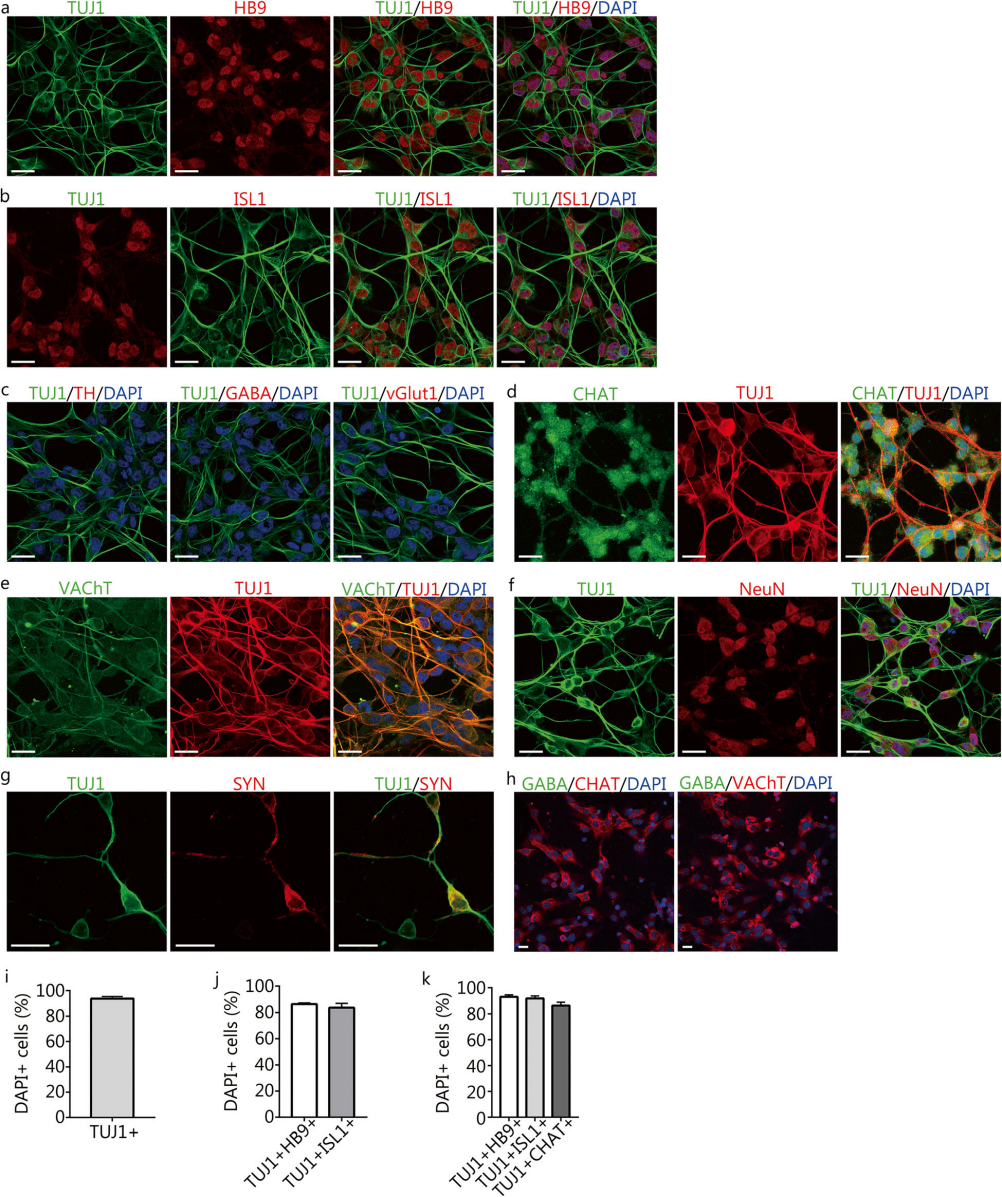
Direct conversion of human astrocytes into MN-like cells using the small-molecule cocktail. **a** and **b** Immunocytochemical analysis of induced neurons for the expression of the neuronal marker TUJ1 and the MN-specific markers HB9 and islet 1 (ISL1) after 10–14 days of chemical induction. Scale bars = 25 μm. **c** Immunostaining assays for the expression of tyrosine hydroxylase (TH), γ-aminobutyric acid (GABA), and vesicular glutamate transporter 1 (vGlut1) in the induced cells. Scale bars = 25 μm. **d**-**g** Immunostaining assays for choline acetyltransferase (CHAT), vesicular acetylcholine transporter (VAChT), neuronal nuclei (NeuN), and synapsin-1 (SYN) after 14 days of chemical induction. **d**, **e**, **f**, scale bars = 25 μm. **g**, scale bar = 50 μm. **h** Expression of CHAT and VAChT in control HA1800 astrocytes. Scale bars = 25 μm. **i** The percentage of TUJ1^+^ cells compared to the that of total DAPI^+^ cells after 2 weeks of induction (mean ± SEM, *n* = 10 randomly selected 20× fields from triplicate samples). **j** The percentages of TUJ1^+^HB9^+^ and TUJ1^+^ISL1^+^ cells compared to the total DAPI^+^ cells after 2 weeks of induction (means ± SEM, *n* = 10 randomly selected 20× fields from triplicate samples). **k** The percentages of TUJ1^+^HB9^+^, TUJ1^+^ISL1^+^, and TUJ1^+^CHAT^+^ cells relative to that of TUJ1^+^ cells induced by small molecules (means ± SEM, *n* = 10 randomly selected 20× fields from triplicate samples)

It has been demonstrated that the ESCs or iPSCs could differentiate into MNs in the presence of RA and Shh/purmorphamine, the activator of the Shh signaling pathway [2, 27, 28]. Therefore, we selected purmorphamine and RA (PR) as the initial cocktail. Treatment with PR did not induce the reprogramming of human astrocytes into neuron-like cells (Additional file 2a). We then added other small molecules that have been reported to induce neuronal conversion from fibroblasts and astrocytes into the initial cocktail, including forskolin, Y-27632, kenpaullone, VPA, Repsox, SB43152, DAPT, dorsomorphin, and LDN193189 [19–23, 25, 29, 30]. After testing different combinations of small molecules, we found that a combination of kenpaullone, forskolin, Y-27632, purmorphamine, and RA (KFYPR) could induce a rapid morphological change in astrocytes from a flat, polygonal shape into a bipolar or multipolar shape as early as 24 h upon chemical treatment (Additional file 2b). Over prolonged induction, the cell bodies became smaller and more compact, with complex neurites (Fig. 1d). Immunostaining analyses revealed that induced cells were positive for TUJ1 after 2 ~ 3 days of induction (Fig. 1e). Moreover, some of these induced neurons expressed the neuronal marker microtubule-associated protein 2 (MAP2) (Fig. 1e). Although a small subset of the control astrocytes expressed TUJ1 in the neurobasal medium without small molecules, they did not alter their morphologies, still expressing high amounts of GFAP and were negative for MAP2 (Fig. 1e). We prolonged the chemical induction until day 7 and then replaced the medium with maturation medium containing forskolin and neurotrophic factors (BDNF, GDNF, and NT3), and after 2 weeks, the induced neurons exhibited complex neurites (Fig. 1d). We then determined the identity of the induced neurons. The immunocytochemical analysis revealed that the induced neurons were positive for the MN markers HB9 and islet 1 (ISL1) (Fig. 2a and b). However, the dopaminergic marker tyrosine hydroxylase (TH), the GABAergic marker γ-aminobutyric acid (GABA), and the glutamatergic marker vesicular glutamate transporter 1 (vGlut1) were undetectable (Fig. 2c). We further examined whether the induced neurons expressed mature MN markers choline acetyltransferase (CHAT) and vesicular acetylcholine transporter (VAChT) by immunostaining assays. The results showed that induced neurons expressed CHAT and VAChT (Fig. 2d and e), whereas the control astrocytes were negative for these markers (Fig. 2h). Furthermore, many of induced neurons were immunopositive for the mature neuronal markers neuronal nuclei (NeuN) and synapsin-1 (SYN) (Fig. 2f and g).

To determine the neuronal conversion efficiency, we calculated the percentages of TUJ1^+^ cells relative to the total DAPI^+^ cells after 2 weeks of induction. Quantitative analysis showed that 93.7 ± 1.6% of the total DAPI^+^ cells were positive for TUJ1 (Fig. 2i), indicating a high astrocyte-neuron conversion efficiency. We then determined the generation efficiency and purity of MN-like cells from human astrocytes by calculating the percentages of the TUJ1^+^HB9^+^, TUJ1^+^ISL1^+^, or TUJ1^+^CHAT^+^ cells to the total DAPI^+^ cells or the induced TUJ1^+^ cells. The numbers of TUJ1^+^HB9^+^ and TUJ1^+^ISL1^+^ cells accounted for approximately 86.5 ± 0.5% and 83.7 ± 1.9% of the total DAPI^+^ cells, respectively (Fig. 2j). These results suggested a relatively high efficiency of generating MN-like cells from human astrocytes. Moreover, the induced MN-like cells appeared highly homogenous, because most of the induced TUJ1^+^ cells were positive for HB9 (93.0 ± 0.8%), ISL1 (91.8 ± 1.1%), or CHAT (86.4 ± 1.5%) (Fig. 2k). Taken together, these results demonstrate the conversion of human astrocytes to MN-like cells with high efficiency. Therefore, we refer to the induced MN-like cells as induced human motor neuron-like cells (hiMNs).

To identify the crucial constituents enabling the neuronal conversion of astrocytes among the chemical cocktails, we removed 1 or 2 small molecule(s) from the cocktail KFYPR in a stepwise manner and observed that K was the most potent inducer of the neuronal morphological change of astrocytes (Additional file 2c). Only 1 or 2 small molecules from the KFYPR cocktail did not significantly convert the astrocytes into neuron-like cells (Additional file 3a and b), whereas this change was observed by adding K to FY, FP, FR, YP, YR, or PR (Additional file 3c). The other three combinations (YPR, FYP, FYR, and FPR) did not appear to induce the change to a neuronal morphology from astrocytes (Additional file 3c). We further investigated whether other GSK-3β inhibitors could replace kenpaullone, such as CHIR99021 (C), a highly selective GSK-3β inhibitor. Although CFYPR could induce astrocytes to form neuron-like cells (Additional file 3d), CFYPR-induced neurons exhibited less neuritic processes compared to the KFYPR-induced neurons. Quantitative analysis revealed the KFYPR-induced cells exhibited longer neurite lengths in comparison to those of CFYPR- cells or FYPR-induced cells (Additional file 3e and f). These results were consistent with previous studies showing that kenpaullone maintains the neuritic processes of normal MNs or ALS-patient MNs [11, 31]. Therefore, kenpaullone was shown to play a crucial role in the neuronal morphological changes in our chemical cocktail.

### hiMN generation does not involve a neural progenitor stage

**Fig. 3:**
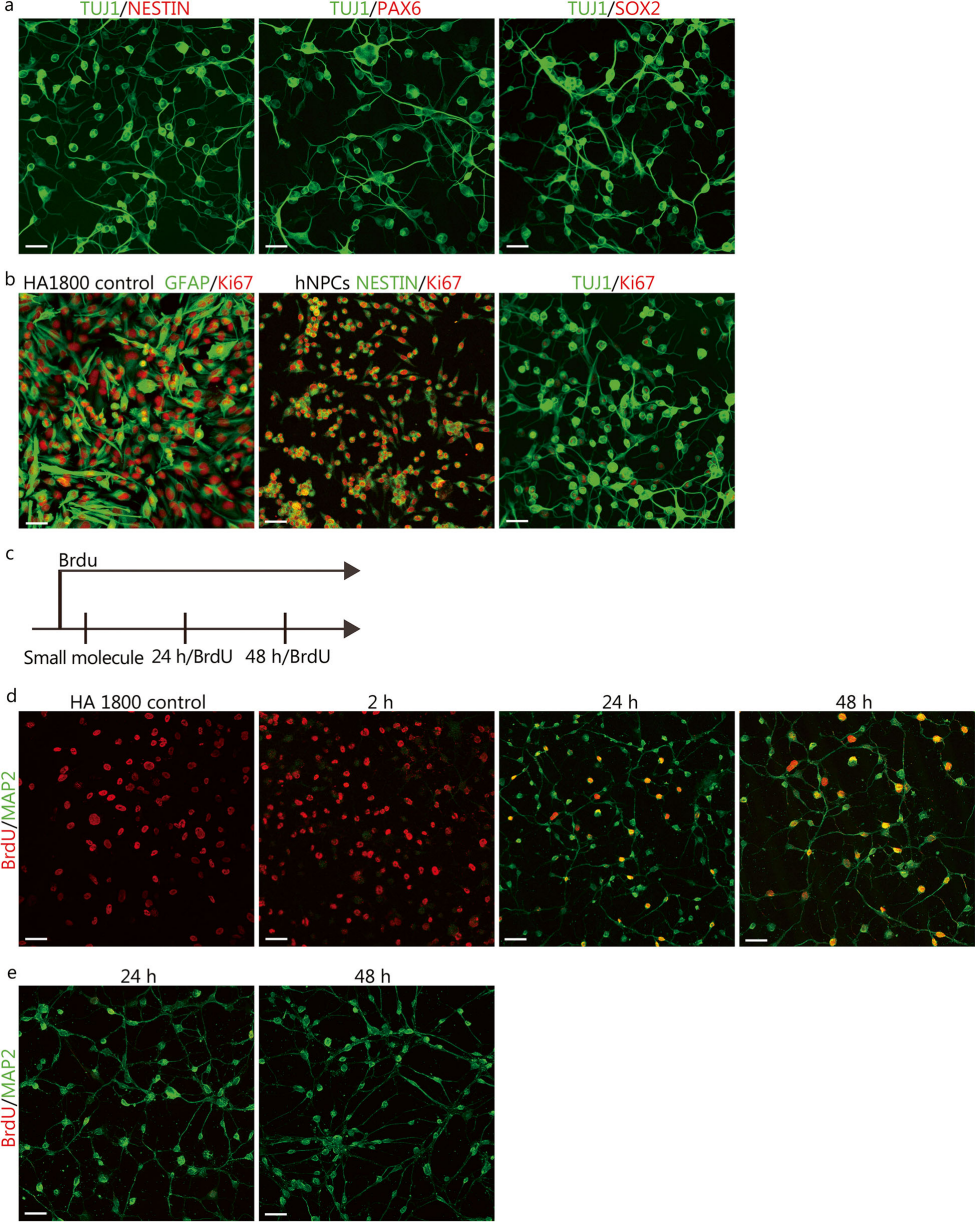
Small molecules directly converted human astrocytes into neurons without passing through a proliferative neural progenitor cell (NPC) stage. **a** Representative images of the expression of the NPC markers NESTIN, PAX6, and SOX2 in human astrocyte-induced neurons after 2–3 days of induction. **b** Representative images of Ki67 expression after chemical induction compared to that observed in HA1800 and human NPCs (hNPCs). **c** Experimental design for the BrdU assay during chemical conversion. Cells were incubated with BrdU at the indicated times. **d** Immunostaining assay for BrdU/MAP2 in human astrocyte-induced neurons that were labeled with BrdU when incubated before small molecule treatment. **e** Immunostaining assay for BrdU/MAP2 in human astrocyte-induced neurons labeled with BrdU at 24 or 48 h after small molecule treatment. All scale bars = 50 μm

To determine whether human astrocytes could dedifferentiate into NPCs during the chemical conversion process, we compared the expression of the NPC markers NESTIN, PAX6, and SOX2 and proliferation marker Ki67 in hiMNs with that of control hNPCs. The immunostaining results showed that the expression of NESTIN, PAX6, and SOX2 was not increased after 2 ~ 3 days of chemical treatment (Fig. 3a). Considering the differences in the morphology of neurons and NPCs, we examined the morphological changes that occurred during the chemical conversion process and did not observe the formation of an NPC-like morphology, which is characterized by a small cell size, bipolar morphology, and the formation of cell clusters (Fig. 1d and Additional file 2b). The number of Ki67-positive cells was significantly decreased after chemical treatment compared to that observed for the initial human astrocytes and was also much lower than that observed for hNPCs (Fig. 3b). These results indicated that no expansion of NPCs occurred after chemical treatment. To further trace whether the cells underwent a proliferative stage during the conversion process (Fig. 3c), we labeled astrocytes with BrdU before chemical treatment. The results showed that many induced cells were positive for BrdU during the chemical induction process and that many subsequent induced neurons were positive for BrdU, indicating that BrdU incorporation was not toxic to the induced cells (Fig. 3d). In contrast, when the induced cells were labeled with BrdU at 24 and 48 h after chemical induction, little or no BrdU incorporation was observed (Fig. 3e). These results showed that no proliferation occurred during chemical induction.

Since the human astrocytes rapidly began exhibiting neuronal shapes as early as 1 day, we attempted to dynamically visualize the process of the morphological changes at the early stage of the astrocyte-neuron conversion. To this end, we transfected human astrocytes with lentiviruses encoding enhanced green fluorescent protein (EGFP) and observed EGFP-positive astrocytes by time-lapse confocal imaging (Additional file 4a). After chemical treatment, the astrocytes rapidly changed from a flat, polygonal morphology to a typical neuronal morphology within 1 day, without cell division (Additional file 4b and Additional file 6, showing a video recording of the early conversion process). Indeed, we observed significant morphological changes as early as 4 h after the addition of small molecules. We observed that most regrown axons or dendrites grew out from the edge of the cell bodies and extended gradually. Furthermore, the newly formed neurites could further bifurcate into small branches, and the cellular bodies become small. We did not observe pre-existing structures of the initial astrocytes that simply remained in place and underwent a remodeling process to form axon-like or dendrite-like structures, because the new axons and dendrites spread out, and the size of the induced cells was much larger than that of their original size. However, we considered that cytoskeletal rearrangements could potentially occur after chemical induction, because the morphologies of the cell bodies of the initial astrocytes displaying flat, polygonal shapes exhibited dynamic changes and turned into relatively round shapes over time. Moreover, some altered parts of cells began to generate new neurites, suggesting that cytoskeletal arrangements may partially contribute to neurite outgrowth. Taken together, these results demonstrate that the chemical induction approach used in the present study does not involve a neural progenitor stage.

### Gene expression of hiMNs

**Fig. 4:**
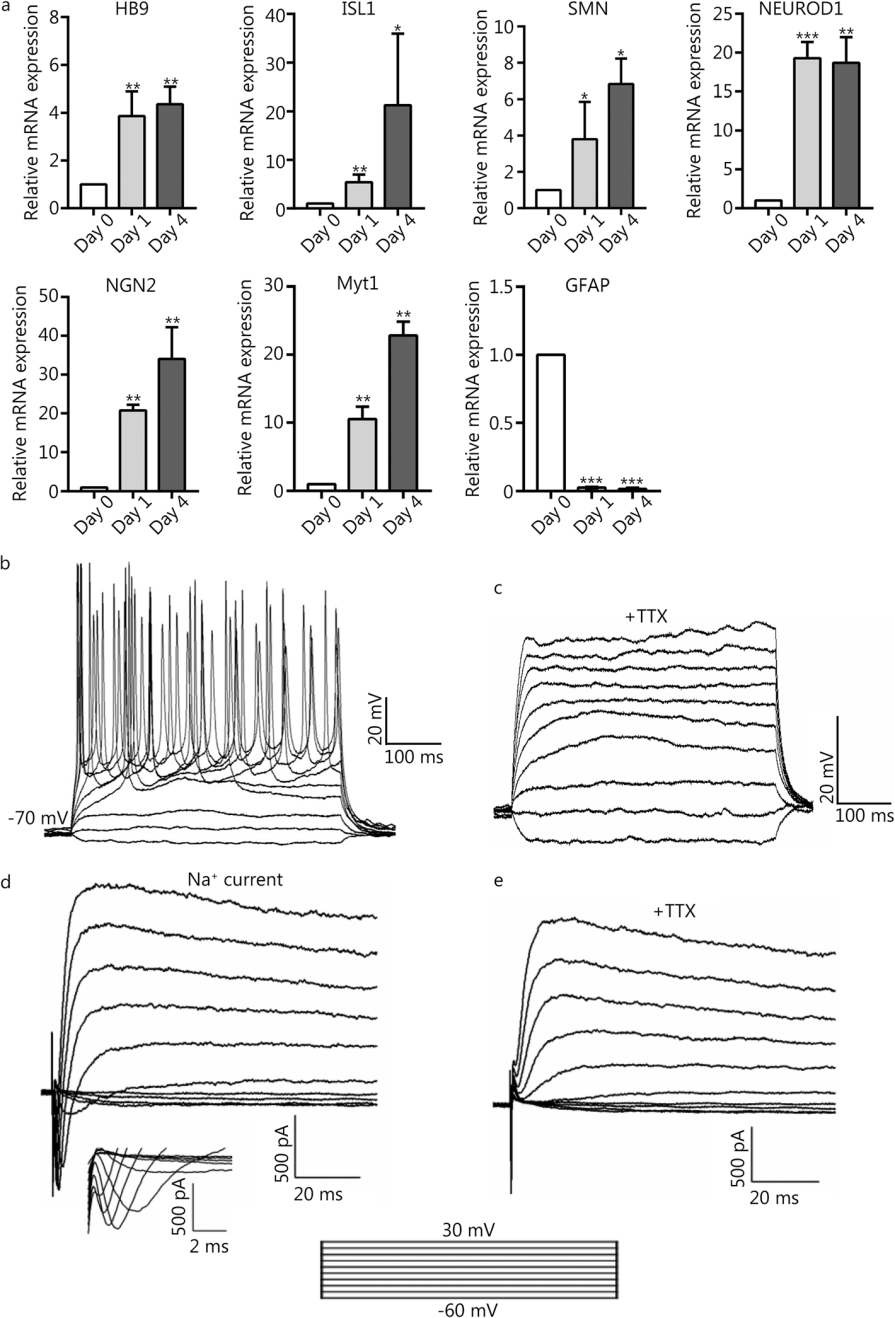
The gene expression profiles and electrophysiological properties of hiMNs. **a** RT-qPCR analysis of mRNA expression levels of genes *HB9*, *ISL1*, *SMN*, *NEUROD1*, *NGN2*, *MYT1*, and *GFAP* during chemical induction. The values are presented as the means ± SEM (*n* = 3. *P < 0.05. **P < 0.01. ***P < 0.001. versus day 0. **b** Current-clamp recordings of hiMN generated from human astrocytes after chemical induction, showing action potentials in response to a depolarizing step current from − 60 to 120 pA (*n* = 6/10, recorded cells). **c** Tetrodotoxin (TTX) could inhibit action potentials. **d** Representative traces of whole-cell current in voltage-clamp mode, showing inward sodium current and outward potassium current (*n* = 5/7, recorded cells). **e** An inward sodium current that was blocked by TTX

We used RT-qPCR to examine the transcriptional changes of the pro-neuronal transcription factor-encoding genes *NEUROD1*, *NGN2*, and *MYTL*; the MN transcription factor-encoding genes *HB9* and *ISL1*; the MN-related gene *SMN*; and the astroglial gene *GFAP* during the chemical induction process. The expression of the genes of *NEUROD1*, *NGN2*, *MYTLl*, *HB9*, *ISL1*, and *SMN* was upregulated, while the expression for *GFAP* was downregulated after chemical treatment (Fig. 4a). Taken together, these results suggested that the small molecules activated the neural transcription program and inhibited astrocytic gene expression.

### hiMNs possess the electrophysiological characteristics of motor neurons

To determine whether hiMNs possess the active membrane properties of neurons, we performed a whole-cell patch-clamp recording of hiMNs 14 ~ 21 days after chemical treatment. Functional neurons are characterized by firing action potentials, and we tested the ability of the hiMNs to fire an action potential in the current-clamp mode. Depolarizing current steps could induce action potentials in these hiMNs (*n* = 6/10, recorded cells) (Fig. 4b), and the action potentials could be blocked by the sodium channel blocker tetrodotoxin (TTX) (Fig. 4c). In the voltage-clamp mode, depolarizing voltage steps triggered large, rapidly inward currents and slow outward currents in hiMNs, consistent with the opening of voltage-activated sodium and potassium channels, respectively (Fig. 4d). Furthermore, the inward current could be blocked by TTX (Fig. 4e). Similar patterns of action potentials and sodium and potassium currents have been detected in human ESC- and iPSC-derived MNs and human fibroblast-derived MNs [9, 11, 12, 32, 33]. Moreover, hiMNs showed electrophysiological properties that were similar to those of the actual MNs derived from healthy mouse spinal cords (Fig. 6a and b). These electrophysiological results suggested that hiMNs can acquire the basic electrophysiological properties of neurons.

### Small molecules induce MN-like cells from astrocytes derived from ALS mouse model

**Fig. 5:**
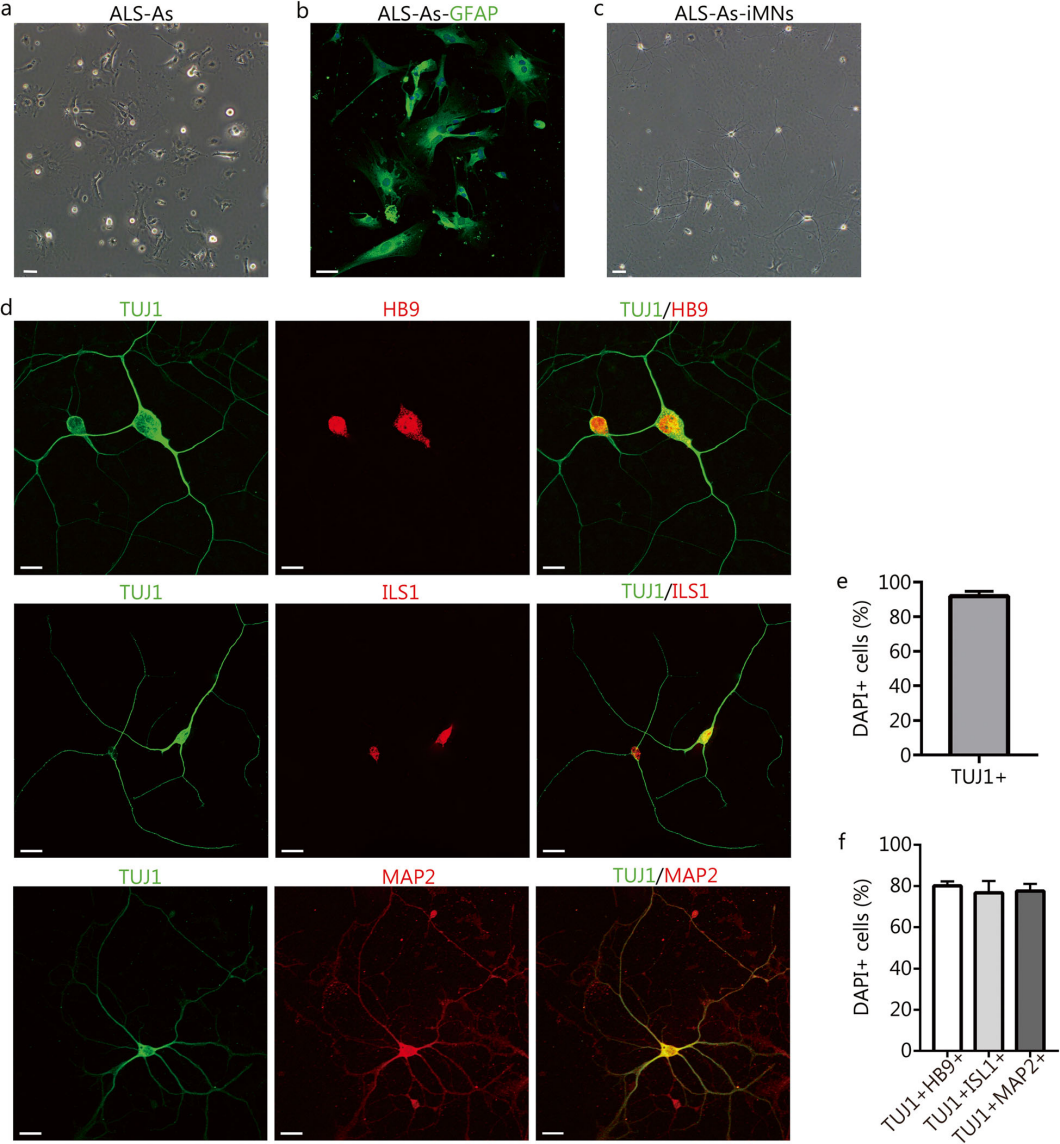
Direct conversion of spinal-cord astrocytes isolated from the adult amyotrophic lateral sclerosis (ALS) mouse model into MN-like cells. **a** Representative images of spinal-cord astrocytes from adult ALS mice (ALS-As). **b** Immunostaining of GFAP expression in ALS-As. **c** Representative images of ALS-As-induced motor neuron-like cells (iMNs) (ALS-As-iMNs). **d** Immunostaining of the neuronal markers TUJ1 and MAP2 and the MN-specific markers HB9 and ISL1 in ALS-As-iMNs. All scale bars = 50 μm. **e** The percentage of TUJ1^+^ cells compared to the total number of DAPI^+^ cells (mean ± SEM, *n* = 10 randomly selected 20× fields from triplicate samples). **f** The percentage of TUJ1^+^HB9^+^ cells to the total number of DAPI^+^ cells (mean ± SEM, *n* = 10 randomly selected 20× fields from triplicate samples)

**Fig. 6:**
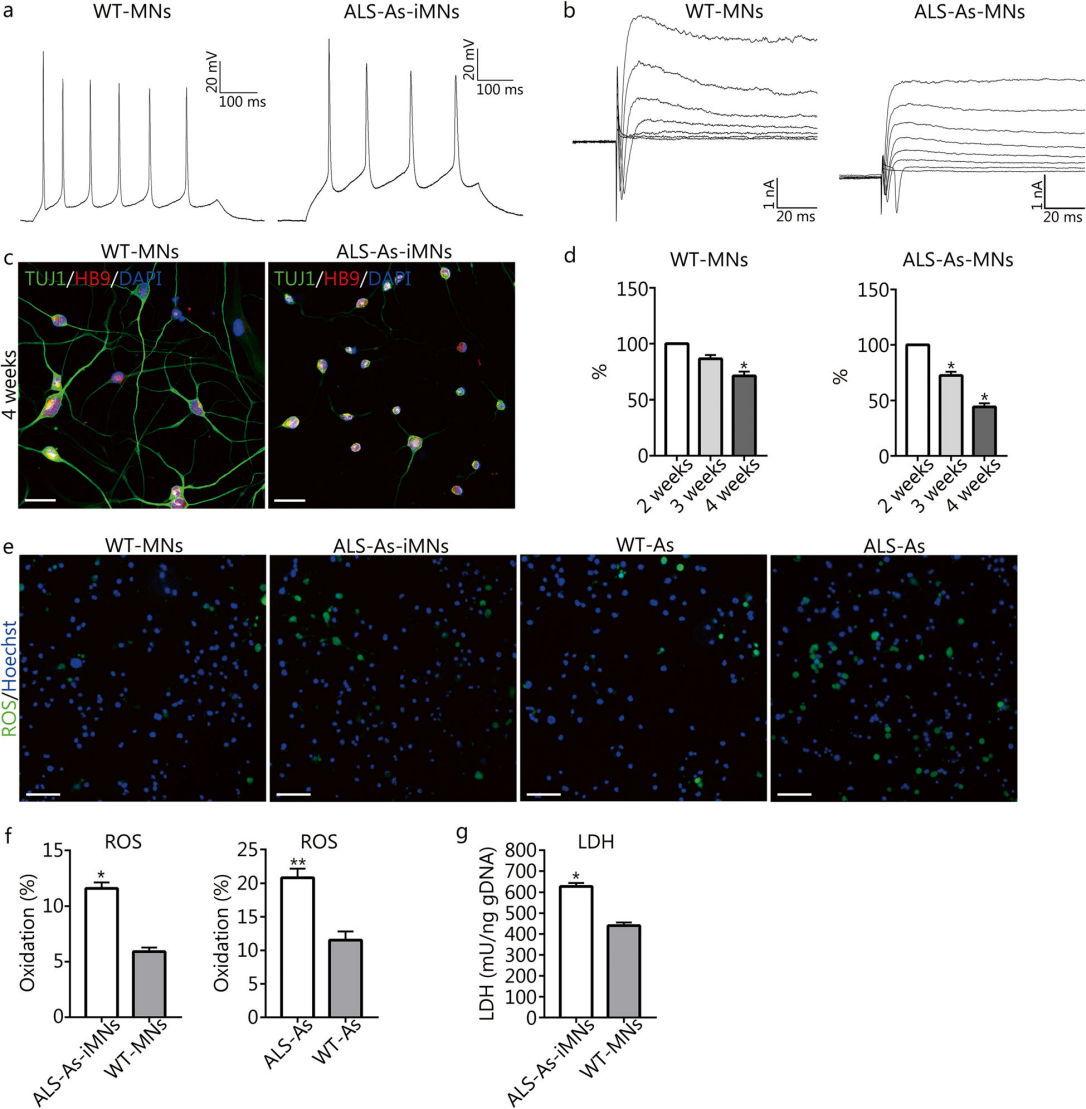
The electrophysiological properties and phenotypic characteristics of ALS-As-iMNs. **a** A current clamp recording showed repetitive firing of action potentials in response to the injection of an 800 ms current pulse (40 pA) in wild type-MNs (WT-MNs) isolated from healthy mouse spinal cords and ALS-As-iMNs (WT-MNs, *n* = 8/12 recorded cells; ALS-As-iMNs, *n* = 6/10 recorded cells). **b** Representative traces of whole-cell current in the voltage-clamp mode in response to depolarizing voltage steps from a holding potential of − 60 at 10-mV increments, showing inward sodium current and outward potassium current (WT-MNs, *n* = 9/12 recorded cells; ALS-As-iMNs, *n* = 8/10 recorded cells). **c** The phenotypes of ALS-As-iMNs and WT-MNs after culturing for 4 weeks. **d** The cell survival rate of ALS-As-iMNs and WT-MNs cultured for 3 and 4 weeks compared to that observed for ALS-As-iMNs cultured for 2 weeks (means ± SEM from three independent experiments, *P < 0.05). **e** and **f** Quantification of the cells producing ROS among ALS astrocytes (ALS-As), ALS-As-iMNs, wild-type astrocytes (WT-As) and WT-MNs isolated from healthy mouse spinal cords (means ± SEM from triplicate samples, **P < 0.01). **g** Colorimetric measurement of LDH (normalized to gDNA) in the supernatants of ALS-As-iMN and WT-MN cultures (means ± SEM from triplicate samples, *P < 0.05)

ALS is a fatal neurological condition, the hallmark of which is the selective and relentless degeneration of motor neurons. Mutant astrocytes in the spinal cords of mice from the SOD1-G93A mouse model of ALS are selectively toxic to motor neurons [25–27], and we attempted to convert the toxic astrocytes into MNs with our chemical cocktail. Astrocytes were isolated from the spinal cords of adult SOD1-G93A mice and cultured in DMEM/F12 medium supplemented with 10% FBS (Fig. 5a). The immunostaining results showed the expression of the astrocytic marker GFAP in the isolated astrocytes (Fig. 5b). After treatment with KFYPR, the mutant astrocytes were converted into MN-like cells, expressing the neuronal marker TUJ1 as well as MN markers (HB9 and ISL1) (Fig. 5c and d). Subsequently, we enumerated the number of TUJ1^+^ and TUJ1^+^HB9^+^ cells to evaluate the conversion efficiency of the mutant astrocytes into MN-like cells. Approximately 91.9 ± 1.6% of mouse astrocytes were induced into TUJ1^+^ neurons (Fig. 5e), and approximately 80.0 ± 2.2% of the induced cells were positive for both TUJ1 and HB9 (Fig. 5f). Then, whole-cell patch-clamp assays were used to examine the electrophysiological properties of the induced neurons and the wild-type (WT)-MNs isolated from healthy mouse spinal cords. The induced neurons could produce action potentials at weeks 2–3 (60.0%, *n* = 10), as shown in Fig. 6a. Interestingly, the patterns of action potentials in the induced neurons were similar to those of our WT-MNs (shown in Fig. 6a) and other MNs reported in previous studies [34–36]. In the voltage-clamp recordings, a series of voltage-steps triggered inward sodium currents and outward potassium currents in the induced neurons, which were similar to those observed in WT-MNs (Fig. 6b). Thus, the chemical cocktail of KFYPR can reprogram the spinal cord astrocyte of the ALS mouse model into MN-like cells (referred to as ALS-As-iMNs).

### ALS-as-iMNs exhibit decreased survival

As SOD1 mutant MNs derived from SOD1-G93A mice display reduced survival, we evaluated how long ALS-As-iMNs could survive in culture. The number of ALS-As-iMNs began to decrease after 2 weeks of induction and continued to decrease over the following weeks (Fig. 6d). In contrast, the number of control WT-MNs decreased less rapidly than that observed for the ALS-As-iMNs (Fig. 6d). We further assessed MN death by measuring the levels of lactate dehydrogenase (LDH), a cytosolic enzyme released after cell death. A higher level of LDH was observed in the culture ALS-As-iMNs than that observed in the WT-MN culture after 4 weeks of induction (Fig. 6g). Taken together, these results indicated that ALS-As-iMNs exhibited a progressive decrease in cell survival.

### Reactive oxygen species in mutant astrocytes and ALS-as-iMNs

It has been reported that mutant SOD1 can increase oxidative stress in astrocytes and MNs and that the toxicity conferred by the SOD1-mutated astrocytes is partly due to an increase in the oxidative stress caused by mutated astrocytes. Accordingly, we investigated the changes in the production of reactive oxygen species (ROS) in the mutant astrocytes (ALS-As) and ALS-As-iMNs. We observed an increase in the number of cells producing ROS in ALS-As compared with that observed in wild-type astrocytes (WT-As) from spinal cords of healthy mice (Fig. 6e and f). Similarly, ALS-As-iMNs exhibited higher ROS production compared to that observed for WT-MNs at 3 weeks (Fig. 6e and f), and the number of cells producing ROS in ALS-As-iMNs was lower than that detected in ALS-As (Fig. 6f). These results suggest that the induction of astrocyte-neuron conversion may reduce the oxidative stress conferred by astrocytes, although ALS-As-iMNs exhibited higher oxidative stress than WT-MNs.

## Discussion

In the present study, we demonstrated that human astrocytes can be chemically converted into hiMNs rapidly and efficiently by small molecules. Importantly, these hiMNs are functional, as evidenced by the characteristic electrophysiological properties of the neurons. In addition, the small-molecule cocktail developed in the present study could induce the formation of MN-like cells from spinal-cord astrocytes of ALS mice.

Previous studies have shown that human ESCs and iPSCs can differentiate into HB9-, ISL1-, and CHAT-positive MNs, although these methods are time-consuming (requiring 1–2 months), complicated (three steps), and inefficient (30–70%) [8, 28, 32, 37–39]. Furthermore, the ethical issues associated with the use of ESCs, the low generation efficiency of iPSCs, and the potential tumor risks of pluripotent stem cells hamper their use. Direct lineage reprogramming provides an alternative approach for generating MNs, with the forced expression of lineage-specific transcription factors having been used to convert mouse and human fibroblasts into spinal MNs [11, 12, 14]. Although the transcription factor-based direct lineage reprogramming avoids the problems associated with ESCs or iPSCs, this method requires genetic manipulation and repeated viral infection, limiting its clinical application. Compared to the above methods, our method of MN derivation from human astrocytes using small molecules is superior in multiple respects, including: 1) a short and easy-to-do process of induction; 2) high efficiency for generating MNs; 3) no genetic or viral manipulation required; and 4) cost-effectiveness. Therefore, our small molecule-based MN induction protocol could potentially be translated into clinical applications.

The conversion process induced by our chemical cocktails was rapid and did not involve an NPC intermediate stage, where small molecule-treated human astrocytes quickly exited the cell cycle. The expression of NPC markers NESTIN, PAX6, and SOX2, were not detected throughout the chemical induction process, which was consistent with the results of previous studies using small molecules to induce neuronal conversion [19, 22, 25]. As neurons are postmitotic, and because the direct reprogramming method bypasses the proliferative stage, the number of neurons obtained simply depends on the initial number of astrocytes used for conversion. Therefore, the yield of neurons that can be obtained by the direct reprogramming approach is often a major concern, limiting its feasibility for cell-based therapies.

The conversion of astrocytes into hiMNs was accompanied by the downregulation of astrocyte-specific genes and the upregulation of endogenous neuronal transcriptional factors. During the conversion process, *NEUROD1* and *NGN2* were the first-wave responsive genes and activated within one day. The proneural transcription factor NGN2 was previously demonstrated to establish neural cells with additional transcriptional factors or small molecules [40]. In addition, NGN2, together with transcription factor combinations (ASCL1, ISL1, NEUROD1, BRN2, HB9, LHX3, and MYT1L) [12, 14] or (SOX11, ISL1, and LHX3) [11] have been reported to directly convert mouse and human fibroblasts into MNs.

We further examined whether KFYPR could induce mouse astrocytes into MN-like cells. As expected, the chemical cocktail efficiently generated MN-like cells from spinal cord astrocytes isolated from the adult ALS mouse model (SOD1-G93A transgenic mice). The ALS-As-iMNs expressed MN markers and possessed the electrophysiological properties similar to those of wild-type MNs isolated from healthy mouse spinal cords. Previous studies have shown that SOD1-mutated MNs display neurodegeneration in vivo and when cultured in vitro over time [34, 41–43]. Similarly, a progressive decrease in cell survival was observed in ALS-As-iMNs after 2–4 weeks. Misfolded SOD1 protein can increase oxidative stress by ROS production in astrocytes and MNs [44, 45]. In the present study, the astrocytes isolated from ALS mouse models exhibited an increased production of ROS compared to wild-type astrocytes. Likewise, the ALS-As-iMNs produced more ROS than wild-type MNs. However, the astrocyte-neuron conversion could reduce the production of ROS due to the lower ROS production observed in ALS-As-iMNs compared to that detected in mutant astrocytes. This phenomenon is important, as astrocyte-neuron conversion can reduce the toxicity (e.g., oxidative stress) conferred by mutant astrocytes. Whether ALS-As-iMNs are toxic toward MNs needs to be investigated in the future. Nevertheless, SOD1-mutated fibroblasts, microglia, cortical neurons, and myocytes do not lead to overt neurotoxicity, demonstrating that SOD1 mutation increases the selective toxicity of astrocytes toward MNs [45–49]. For this reason, we think that once mutant astrocytes lose the identity of astrocytes, they will lose or attenuate their toxicity toward MNs.

Although ALS-As-iMNs carrying SOD1 mutations still display neurodegeneration, it remains unclear whether MN-like cells reprogrammed from astrocytes of sporadic ALS patients will be vulnerable to undergoing neurodegeneration. Given that spinal cord astrocytes are selectively toxic toward spinal MNs in familial and sporadic ALS [47–51], the direct conversion of ALS astrocytes into MN-like cells may reduce their neurotoxicity. In addition, the conversion of healthy astrocytes to functional MNs may provide promising sources for cell-based therapies. Because astrocyte activation after traumatic spinal cord injury (SCI) leads to glial scars that inhibit the MN and axon regeneration [52], the direct conversion of resident reactive astrocytes into MN-like cells will promote functional recovery after SCI. Thus, the small molecule-based generation of MNs from spinal cord astrocytes will be beneficial for patients with neurodegenerative motor neuron diseases or SCI.

## Conclusions

In summary, the results of our study showed the efficacy of a small molecule-based approach for MN generation from human and mouse astrocytes. Our hiMNs may be applicable for use in cell replacement therapies for patients with motor neuron diseases or SCI. The MN-like cells induced by reprogramming mutant astrocytes from the ALS mouse model may act as cellular models for ALS diseases and drug screening. Furthermore, the small-molecule cocktail developed in the present study may hopefully be translated into a therapy to directly convert resident spinal-cord astrocytes into MNs in situ to participate in the repair and regeneration of motor neurons in spinal cords.

## Supplementary information

**Additional file 1.** Characterization of cultured human astrocytes. (a) Immunocytochemical results showing the expression of the NPC markers NESTIN, PAX6, and SOX2 on cultured human astrocytes. Cultured human astrocytes did not express NPC markers, whereas human NPCs obviously expressed NPC markers. Representative images of three independent experiments. Scale bar = 50 μm. (b) Immunocytochemical results showing the expression of the neuronal markers TUJ1 and MAP2 and oligodendrocyte marker O4 on human astrocytes after culturing in neuronal differentiation medium for three weeks. Scale bar = 50 μm. (c) Flow cytometry analysis of CD44 expression on cultured human astrocytes. The quantitative percentage of CD44-expressing cells were shown in three independent experiments. (d) The ability of cultured human astrocytes to form neurospheres. Cultured human astrocytes did not form neurospheres after being cultured in the medium to induce neurosphere formation. In contrast, human NPCs formed neurospheres under the same culture condition, as a positive control. Representative images of three independent experiments. Scale bars = 50 μm.

**Additional file 2.** The morphological changes after treatment with small molecules. (a) Representative images of control HA1800 astrocytes and PR-treated astrocytes after 5 days of induction. (b) The morphological changes after KFYPR treatment at the early stages. (c) The morphological changes after treatment with different combinations of four small molecules (KFPR, KFYR, KYPR, KFYP, and FYPR) after 5 days of induction. Scale bars = 50 μm.

**Additional file 3. **Morphological changes of human astrocytes after treatment with different small molecules. (a) Morphological changes induced by treatment with only one molecule [purmorphamine (P), retinoic acid (R), forskolin (F), Y-27632 (Y), kenpaullone (K)] after 5 days of induction. (b) Morphological changes induced by treatment with different combinations of 2 small molecules (PR, FR, FY, FP, YP, YR, KP, KR, KF, and KY) after 5 days of induction. (c) Morphological changes induced by treatment with three small molecules (KPR, KFR, KYR, KYP, KFP, KFY, YPR, FYP, FYR, and FPR) after 5 days of induction. (d) Morphological changes induced by treatment with CFYPR (CHIR99021, forskolin, Y-27632, purmorphamine, and retinoic acid) after 5 days of induction. Scale bars = 100 μm. (e) Immunostaining for TUJ1 in KFYPR-, CFYPR-, and FYPR-induced cells. (f) Quantification of the relative neurite lengths of KFYPR- and FYPR-induced cells compared with that observed for CFYPR-induced cells (*n* = 30 neurons, mean ± SEM from triplicate samples, *P < 0.05).

**Additional file 4.** Small molecules induce a rapid morphological change of human astrocytes into neuron-like cells. (a) Experimental design. (b) Time-lapse live-cell imaging after treatment with small molecules within 24 h. The white arrow indicates a cell rapidly changing its shape into one with neuronal morphology.

**Additional file 5.** Primer lists for RT-qPCR.

**Additional file 6.** Time-lapse live cell imaging from 0 to 24 h after the chemical treatment.

## Data Availability

The data and materials used in the current study are all available from the corresponding author upon reasonable request.

## Abbreviations

ALS: Amyotrophic lateral sclerosis
ESCs: Embryonic stem cells
iPSCs: Induced pluripotent stem cells
MAP2: Microtubule-associated protein 2
MNs: Motor neurons
NPCs: Neural progenitor cells
RT-qPCR: Quantitative reverse transcription polymerase chain reaction
SCI: Spinal cord injury
TTX: Tetrodotoxin
