# Correction to: Efficient and rapid conversion of human astrocytes and ALS mouse model spinal cord astrocytes into motor neuron-like cells by defined small molecules

**DOI:** 10.1186/s40779-021-00312-9

**Published:** 2021-04-06

**Authors:** An-Dong Zhao, Hua Qin, Meng-Li Sun, Kui Ma, Xiao-Bing Fu

**Affiliations:** 1 Tianjin Medical University, Tianjin, 300070 China; 2 Research Center for Tissue Repair and Regeneration affiliated to the Medical Innovation Research Division and 4th Medical Center, PLA General Hospital and PLA Medical College, 28 Fu Xing Road, Haidian District, Beijing, 100853 P. R. China; 3 PLA Key Laboratory of Tissue Repair and Regenerative Medicine and Beijing Key Research Laboratory of Skin Injury, Repair and Regeneration, Beijing, 100048 China; 4 Research Unit of Trauma Care, Tissue Repair and Regeneration, Chinese Academy of Medical Sciences, Beijing, 100048 China

**Correction to: Military Med Res 7, 42 (2020)**

**https://doi.org/10.1186/s40779-020-00271-7**

In the original publication of this article [1], Figs. 2 and 4, additional file figure 2, additional file figure 3, additional file figure 4 are incorrect, the correct figures are given below. The original publication has been corrected.

**Fig. 2:**
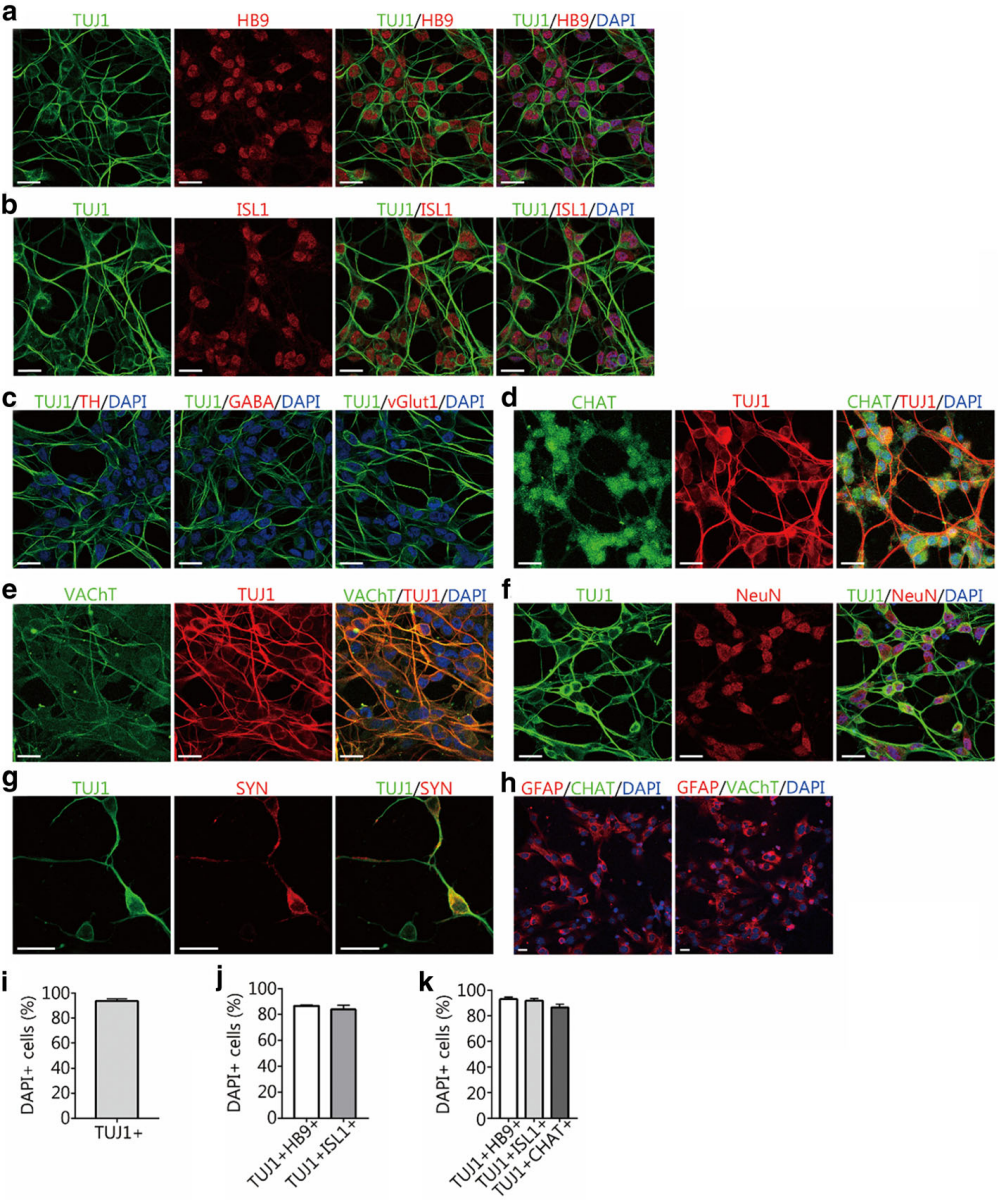
Direct conversion of human astrocytes into MN-like cells using the small-molecule cocktail. **a** and **b** Immunocytochemical analysis of induced neurons for the expression of the neuronal marker TUJ1 and the MN-specific markers HB9 and islet 1 (ISL1) after 10–14 days of chemical induction. Scale bars = 25 μm. **c** Immunostaining assays for the expression of tyrosine hydroxylase (TH), γ-aminobutyric acid (GABA), and vesicular glutamate transporter 1 (vGlut1) in the induced cells. Scale bars = 25 μm. **d**-**g** Immunostaining assays for choline acetyltransferase (CHAT), vesicular acetylcholine transporter (VAChT), neuronal nuclei (NeuN), and synapsin-1 (SYN) after 14 days of chemical induction. **d**, **e**, **f**, scale bars = 25 μm. g, scale bar = 50 μm. **h** Expression of CHAT and VAChT in control HA1800 astrocytes. Scale bars = 25 μm. **i** The percentage of TUJ1^+^ cells compared to the that of total DAPI^+^ cells after 2 weeks of induction (mean ± SEM, *n* = 10 randomly selected 20× fields from triplicate samples). **j** The percentages of TUJ1^+^HB9^+^ and TUJ1^+^ISL1^+^ cells compared to the total DAPI^+^ cells after 2 weeks of induction (means ± SEM, *n* = 10 randomly selected 20× fields from triplicate samples). **k** The percentages of TUJ1^+^HB9^+^, TUJ1^+^ISL1^+^, and TUJ1^+^CHAT^+^ cells relative to that of TUJ1^+^ cells induced by small molecules (means ± SEM, n = 10 randomly selected 20× fields from triplicate samples)

**Fig. 4:**
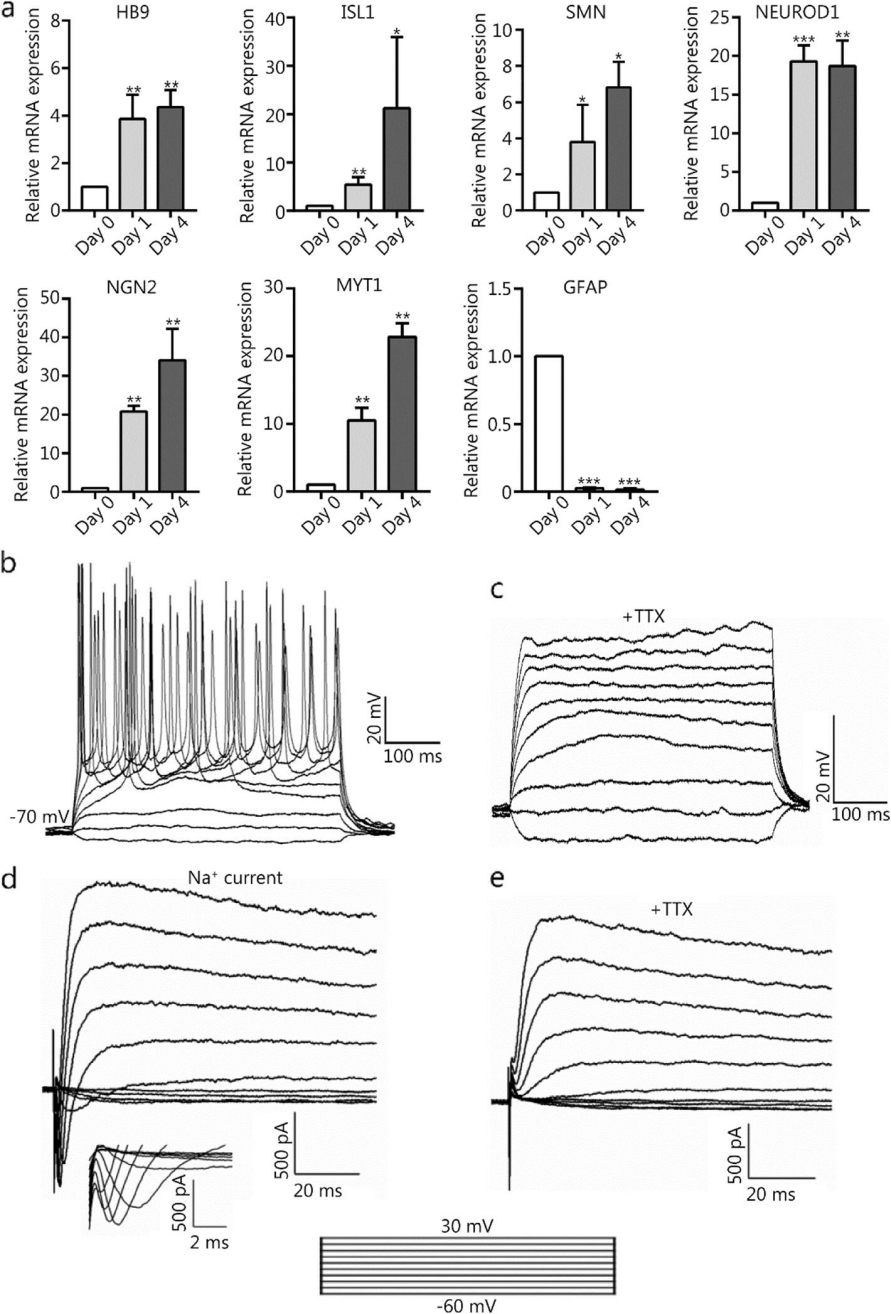
The gene expression profiles and electrophysiological properties of hiMNs. **a** RT-qPCR analysis of mRNA expression levels of genes HB9, ISL1, SMN, NEUROD1, NGN2, MYT1, and GFAP during chemical induction. The values are presented as the means ± SEM (*n* = 3. *P < 0.05. **P < 0.01. ***P < 0.001. versus day 0. **b** Current-clamp recordings of hiMN generated from human astrocytes after chemical induction, showing action potentials in response to a depolarizing step current from − 60 to 120 pA (*n* = 6/10, recorded cells). **c** Tetrodotoxin (TTX) could inhibit action potentials. **d** Representative traces of whole-cell current in voltage-clamp mode, showing inward sodium current and outward potassium current (*n* = 5/7, recorded cells). **e** An inward sodium current that was blocked by TTX

## Supplementary Information

**Additional file 2.** The morphological changes after treatment with small molecules. (a) Representative images of control HA1800 astrocytes and PR-treated astrocytes after 5 days of induction. (b) The morphological changes after KFYPR treatment at the early stages. (c) The morphological changes after treatment with different combinations of four small molecules (KFPR, KFYR, KYPR, KFYP, and FYPR) after 5 days of induction. Scale bars = 50 μm

**Additional file 3. **Morphological changes of human astrocytes after treatment with different small molecules. (a) Morphological changes induced by treatment with only one molecule [purmorphamine (P), retinoic acid (R), forskolin (F), Y-27632 (Y), kenpaullone (K)] after 5 days of induction. (b) Morphological changes induced by treatment with different combinations of 2 small molecules (PR, FR, FY, FP, YP, YR, KP, KR, KF, and KY) after 5 days of induction. (c) Morphological changes induced by treatment with three small molecules (KPR, KFR, KYR, KYP, KFP, KFY, YPR, FYP, FYR, and FPR) after 5 days of induction. (d) Morphological changes induced by treatment with CFYPR (CHIR99021, forskolin, Y-27632, purmorphamine, and retinoic acid) after 5 days of induction. Scale bars = 100 μm. (e) Immunostaining for TUJ1 in KFYPR-, CFYPR-, and FYPR-induced cells. (f) Quantification of the relative neurite lengths of KFYPR- and FYPR-induced cells compared with that observed for CFYPR-induced cells (*n* = 30 neurons, mean ± SEM from triplicate samples, *P < 0.05)

**Additional file 4.** Small molecules induce a rapid morphological change of human astrocytes into neuron-like cells. (a) Experimental design. (b) Time-lapse live-cell imaging after treatment with small molecules within 24 h. The white arrow indicates a cell rapidly changing its shape into one with neuronal morphology

## References

[1] Zhao AD (2020). Efficient and rapid conversion of human astrocytes and ALS mouse model spinal cord astrocytes into motor neuron-like cells by defined small molecules. Military Med Res.

