# Supplementary figures and images for: Efficient and rapid conversion of human astrocytes and ALS mouse model spinal cord astrocytes into motor neuron-like cells by defined small molecules
Source: Mil Med Res. 2020 Sep 6;7:42. doi: 10.1186/s40779-020-00271-7 (PMC7487818; doi:10.1186/s40779-020-00271-7)

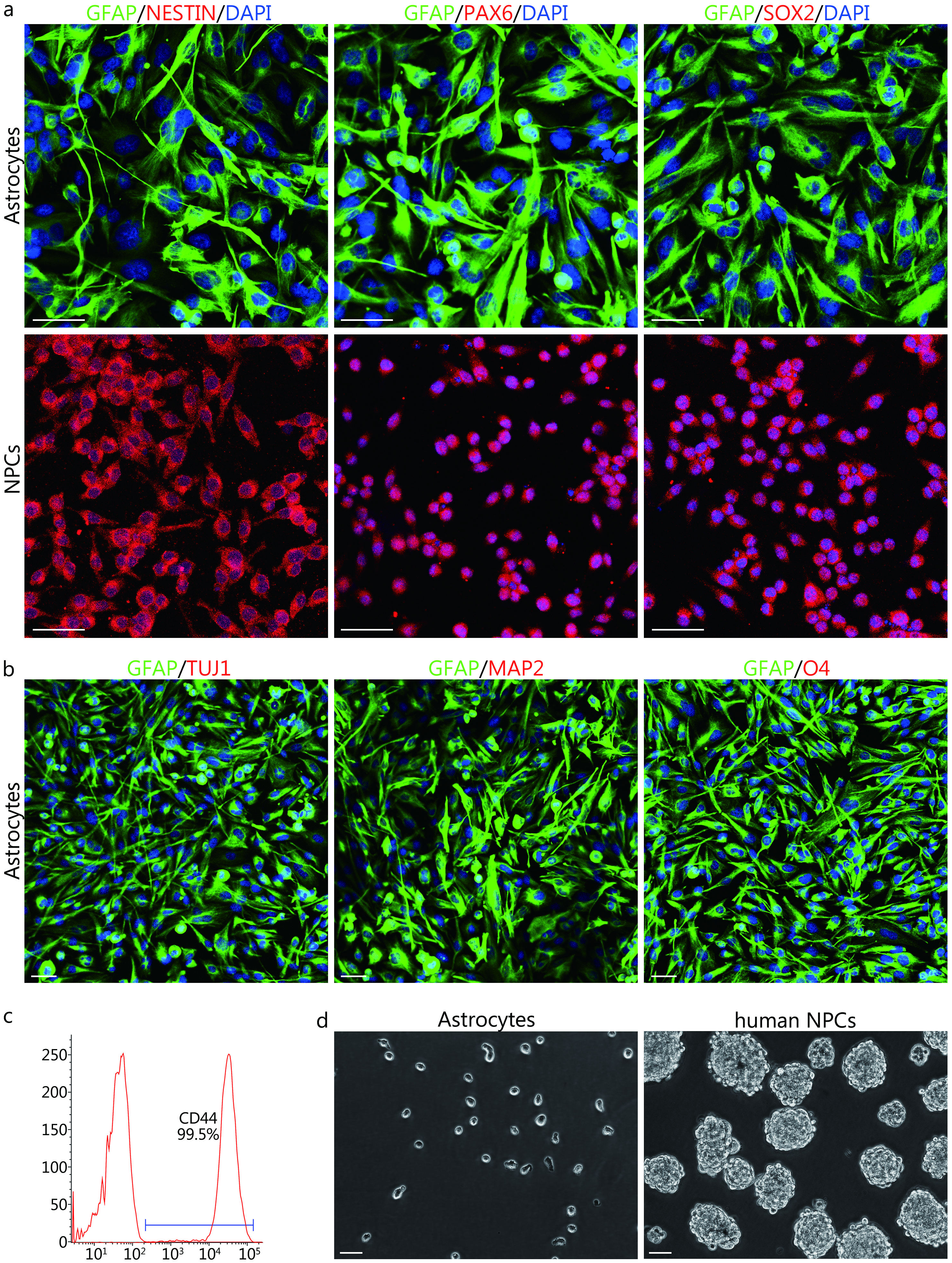

Supplement: Supplementary file 1 — Additional file 1. Characterization of cultured human astrocytes. (a) Immunocytochemical results showing the expression of the NPC markers NESTIN, PAX6, and SOX2 on cultured human astrocytes. Cultured human astrocytes did not express NPC markers, whereas human NPCs obviously expressed NPC markers. Representative images of three independent experiments. Scale bar = 50 μm. (b) Immunocytochemical results showing the expression of the neuronal markers TUJ1 and MAP2 and oligodendrocyte marker O4 on human astrocytes after culturing in neuronal differentiation medium for three weeks. Scale bar = 50 μm. (c) Flow cytometry analysis of CD44 expression on cultured human astrocytes. The quantitative percentage of CD44-expressing cells were shown in three independent experiments. (d) The ability of cultured human astrocytes to form neurospheres. Cultured human astrocytes did not form neurospheres after being cultured in the medium to induce neurosphere formation. In contrast, human NPCs formed neurospheres under the same culture condition, as a positive control. Representative images of three independent experiments. Scale bars = 50 μm. [file 40779_2020_271_MOESM1_ESM.tif]

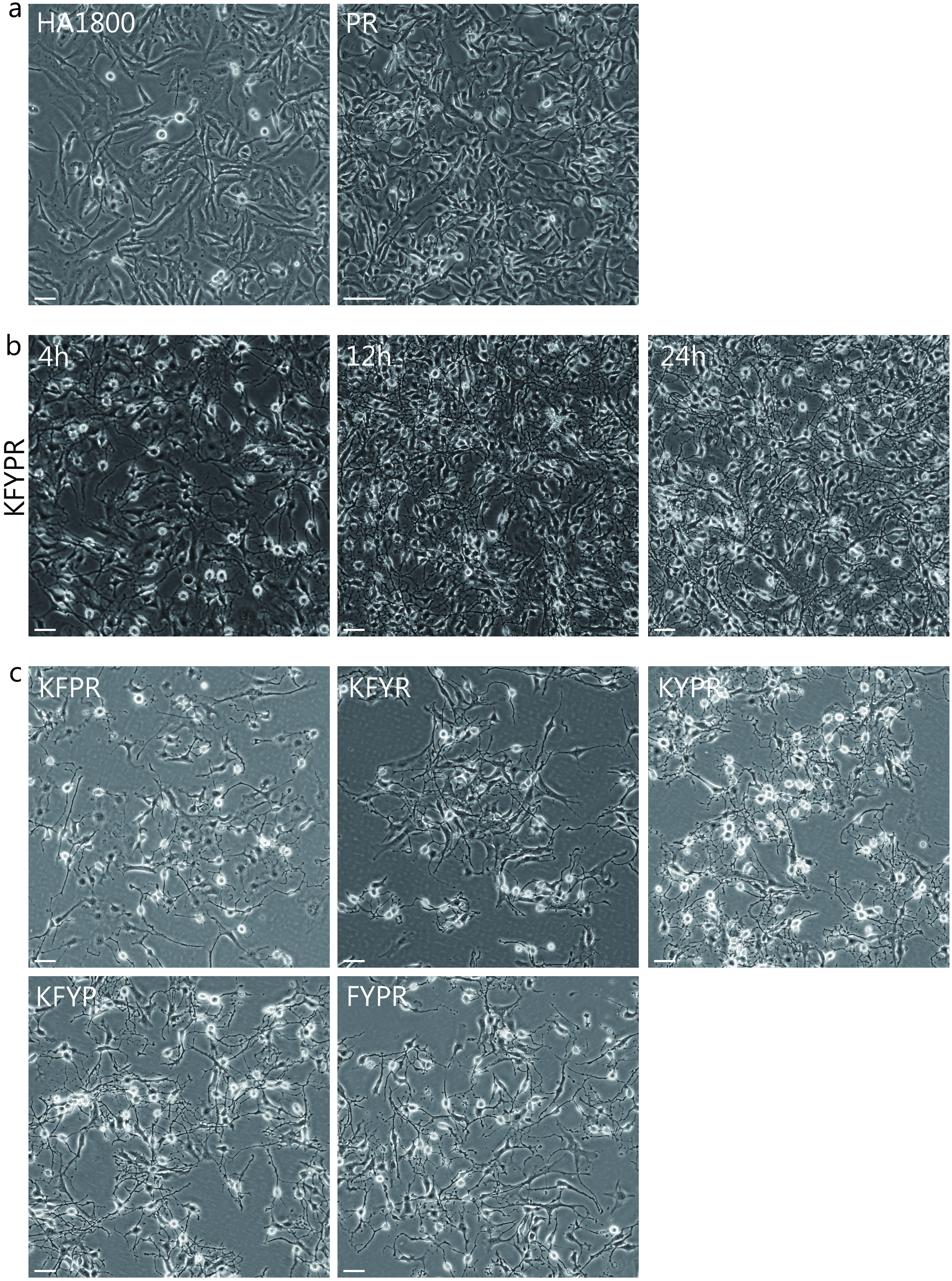

Supplement: Supplementary file 2 — Additional file 2. The morphological changes after treatment with small molecules. (a) Representative images of control HA1800 astrocytes and PR-treated astrocytes after 5 days of induction. (b) The morphological changes after KFYPR treatment at the early stages. (c) The morphological changes after treatment with different combinations of four small molecules (KFPR, KFYR, KYPR, KFYP, and FYPR) after 5 days of induction. Scale bars = 50 μm. [file 40779_2020_271_MOESM2_ESM.tif]

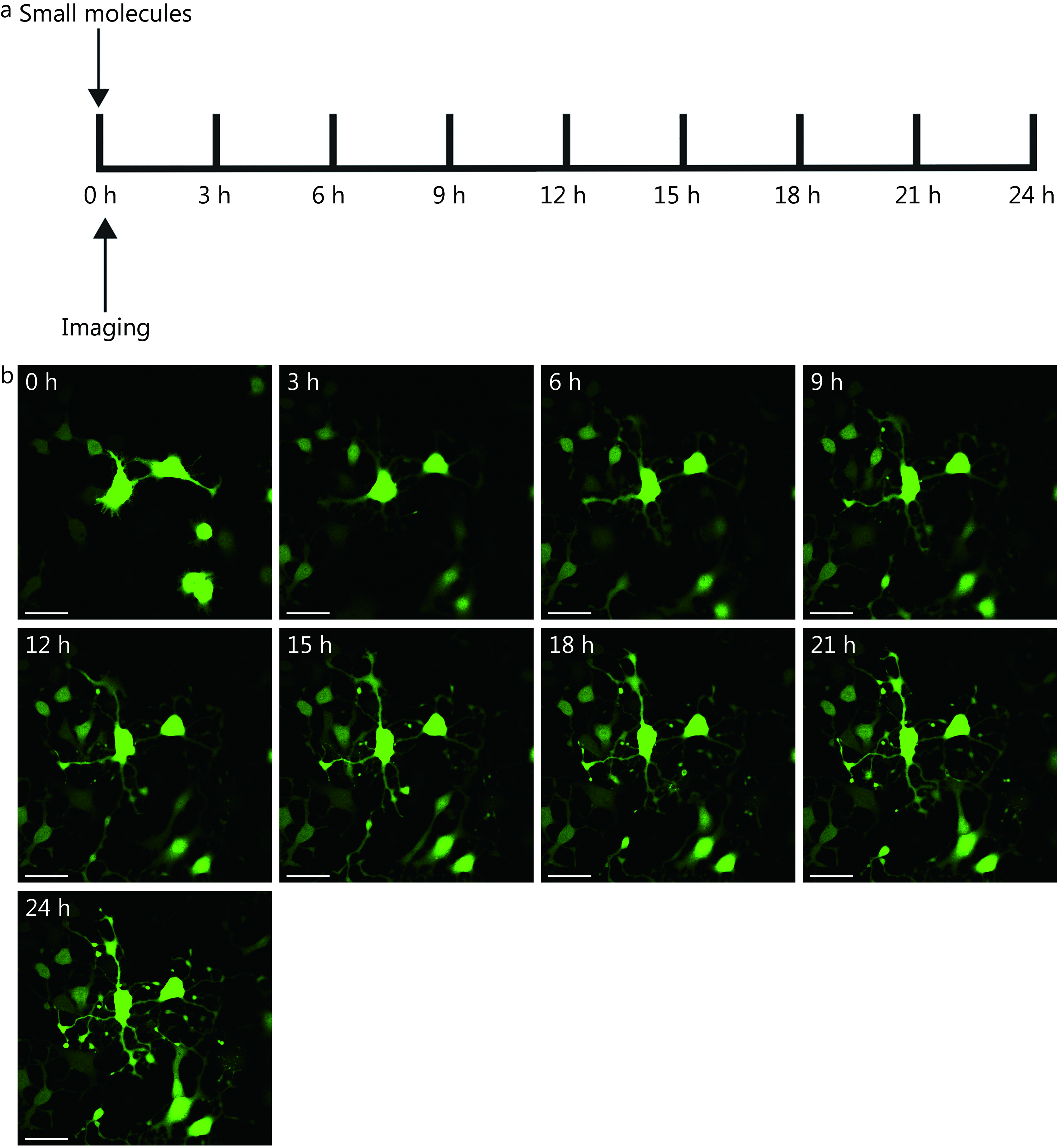

Supplement: Supplementary file 4 — Additional file 4. Small molecules induce a rapid morphological change of human astrocytes into neuron-like cells. (a) Experimental design. (b) Time-lapse live-cell imaging after treatment with small molecules within 24 h. The white arrow indicates a cell rapidly changing its shape into one with neuronal morphology. [file 40779_2020_271_MOESM4_ESM.tif]

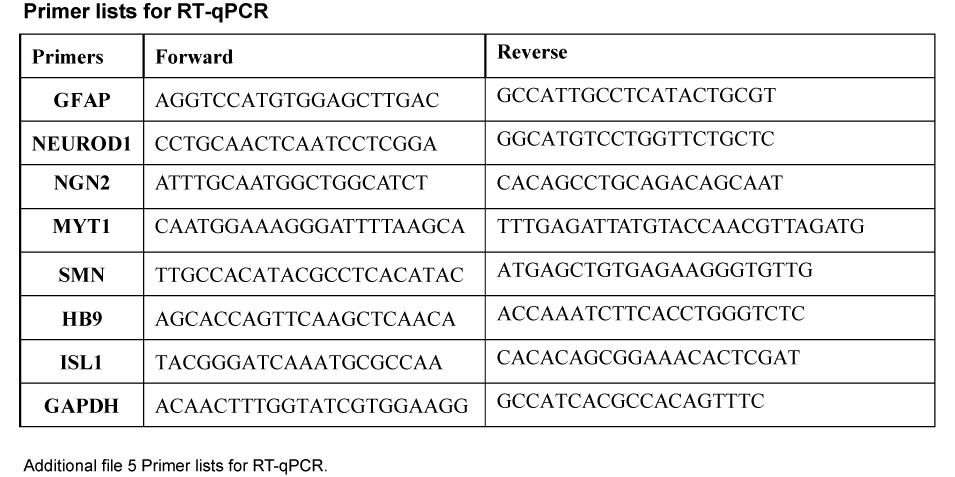

Supplement: Supplementary file 5 — Additional file 5. Primer lists for RT-qPCR. [file 40779_2020_271_MOESM5_ESM.tif]
