# Supplementary figures and images for: Correction to: Efficient and rapid conversion of human astrocytes and ALS mouse model spinal cord astrocytes into motor neuron-like cells by defined small molecules
Source: Mil Med Res. 2021 Apr 6;8:24. doi: 10.1186/s40779-021-00312-9 (PMC8022435; doi:10.1186/s40779-021-00312-9)

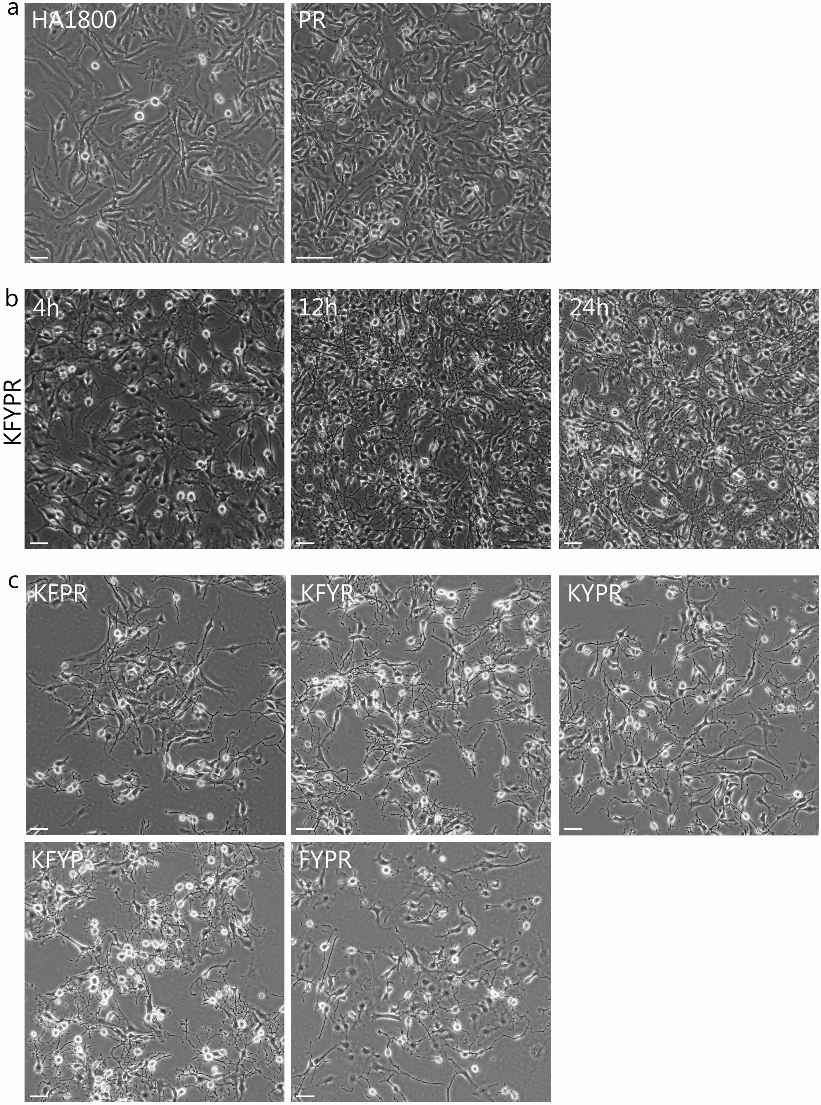

Supplement: Supplementary file 1 — Additional file 2. The morphological changes after treatment with small molecules. (a) Representative images of control HA1800 astrocytes and PR-treated astrocytes after 5 days of induction. (b) The morphological changes after KFYPR treatment at the early stages. (c) The morphological changes after treatment with different combinations of four small molecules (KFPR, KFYR, KYPR, KFYP, and FYPR) after 5 days of induction. Scale bars = 50 μm [file 40779_2021_312_MOESM2_ESM.docx]

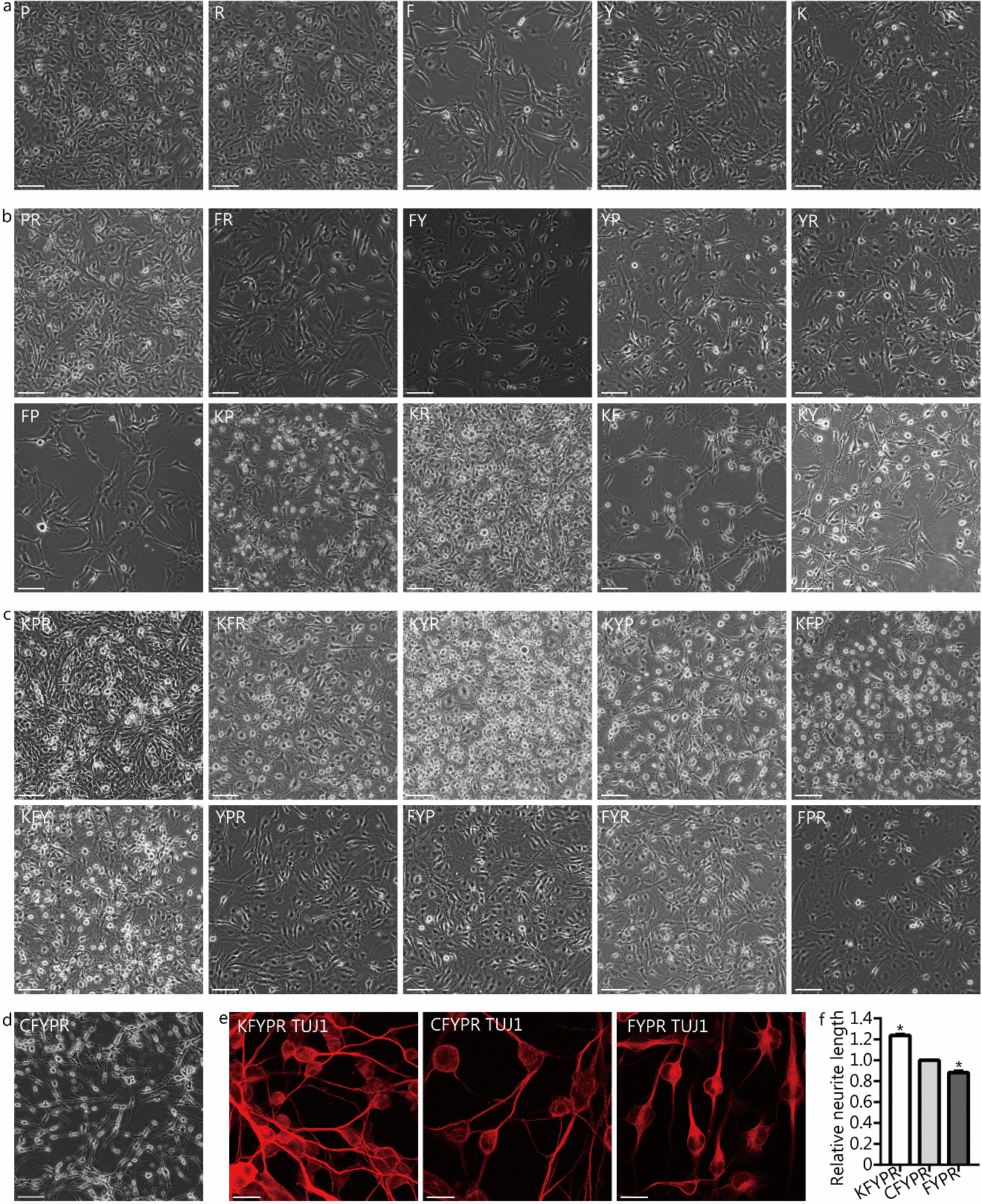

Supplement: Supplementary file 2 — Additional file 3. Morphological changes of human astrocytes after treatment with different small molecules. (a) Morphological changes induced by treatment with only one molecule [purmorphamine (P), retinoic acid (R), forskolin (F), Y-27632 (Y), kenpaullone (K)] after 5 days of induction. (b) Morphological changes induced by treatment with different combinations of 2 small molecules (PR, FR, FY, FP, YP, YR, KP, KR, KF, and KY) after 5 days of induction. (c) Morphological changes induced by treatment with three small molecules (KPR, KFR, KYR, KYP, KFP, KFY, YPR, FYP, FYR, and FPR) after 5 days of induction. (d) Morphological changes induced by treatment with CFYPR (CHIR99021, forskolin, Y-27632, purmorphamine, and retinoic acid) after 5 days of induction. Scale bars = 100 μm. (e) Immunostaining for TUJ1 in KFYPR-, CFYPR-, and FYPR-induced cells. (f) Quantification of the relative neurite lengths of KFYPR- and FYPR-induced cells compared with that observed for CFYPR-induced cells (n = 30 neurons, mean ± SEM from triplicate samples, *P < 0.05) [file 40779_2021_312_MOESM3_ESM.docx]

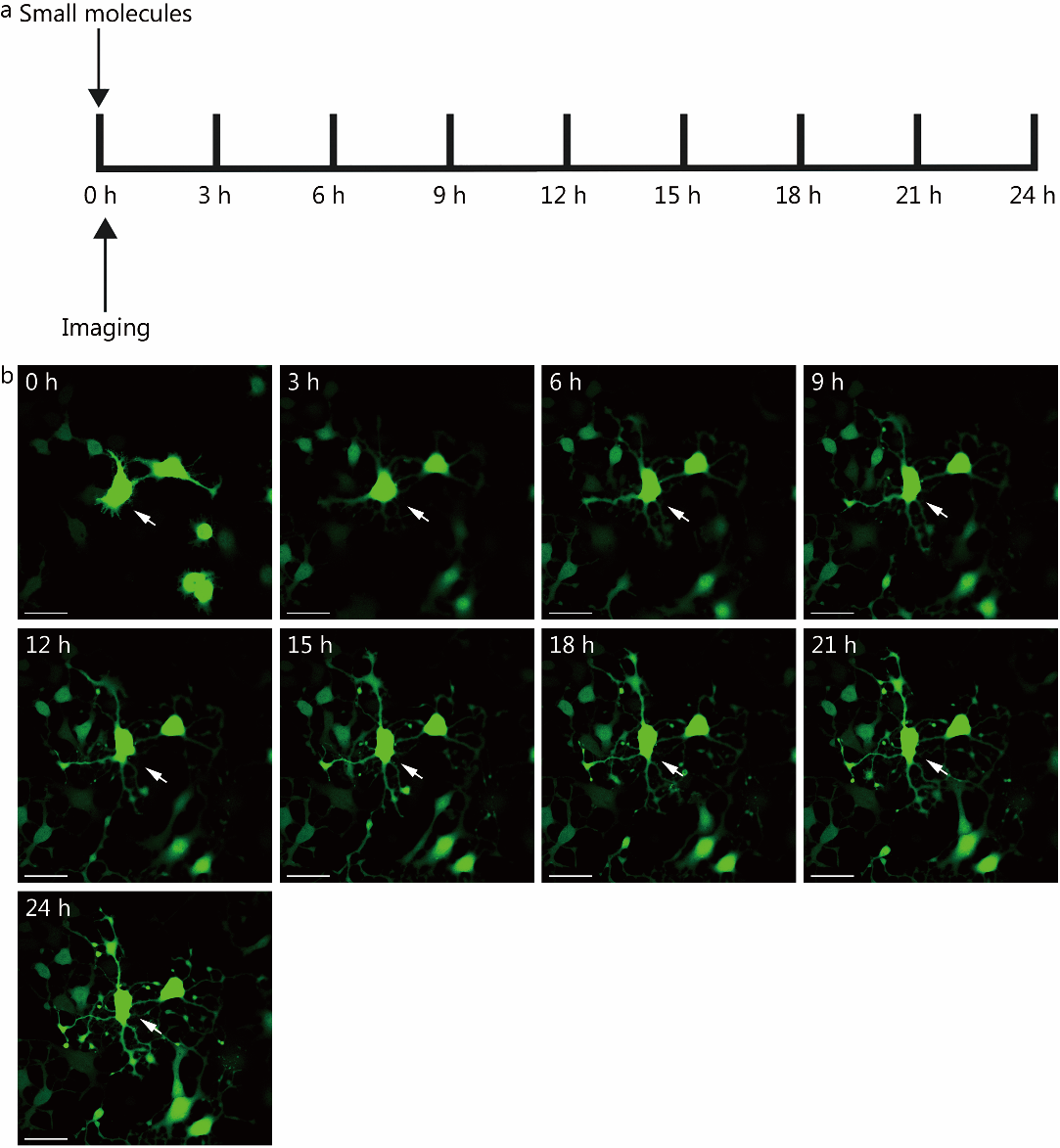

Supplement: Supplementary file 3 — Additional file 4. Small molecules induce a rapid morphological change of human astrocytes into neuron-like cells. (a) Experimental design. (b) Time-lapse live-cell imaging after treatment with small molecules within 24 h. The white arrow indicates a cell rapidly changing its shape into one with neuronal morphology [file 40779_2021_312_MOESM4_ESM.docx]
